# Wetting and Spreading Behavior of Axisymmetric Compound Droplets on Curved Solid Walls Using Conservative Phase Field Lattice Boltzmann Method

**DOI:** 10.3390/e26020172

**Published:** 2024-02-17

**Authors:** Yue Wang, Jun-Jie Huang

**Affiliations:** 1 College of Aerospace Engineering, Chongqing University, Chongqing 400044, China; 202131021038@stu.cqu.edu.cn; 2 Chongqing Key Laboratory of Heterogeneous Material Mechanics, Chongqing University, Chongqing 400044, China

**Keywords:** compound droplet, lattice Boltzmann method, droplet separation, wetting, conservative phase field, curved wall

## Abstract

Compound droplets have received increasing attention due to their applications in many several areas, including medicine and materials. Previous works mostly focused on compound droplets on planar surfaces and, as such, the effects of curved walls have not been studied thoroughly. In this paper, the influence of the properties of curved solid wall (including the shape, curvature, and contact angle) on the wetting behavior of compound droplets is explored. The axisymmetric lattice Boltzmann method, based on the conservative phase field formulation for ternary fluids, was used to numerically study the wetting and spreading of a compound droplet of the Janus type on various curved solid walls at large density ratios, focusing on whether the separation of compound droplets occurs. Several types of wall geometries were considered, including a planar wall, a concave wall with constant curvature, and a convex wall with fixed or variable curvature (specifically, a prolate or oblate spheroid). The effects of surface wettability, interfacial angles, and the density ratio (of droplet to ambient fluid) on the wetting process were also explored. In general, it was found that, under otherwise identical conditions, droplet separation tends to happen more likely on more hydrophilic walls, under larger interfacial angles (measured inside the droplet), and at larger density ratios. On convex walls, a larger radius of curvature of the surface near the droplet was found to be helpful to split the Janus droplet. On concave walls, as the radius of curvature increases from a small value, the possibility to observe droplet separation first increases and then decreases. Several phase diagrams on whether droplet separation occurs during the spreading process were produced for different kinds of walls to illustrate the influences of various factors.

## 1. Introduction

The movement of compound droplets composed of different components occurs commonly in nature and engineering applications. Due to the potential applications in manufacturing of inkjet printing [1], drug delivery [2], microfluidic preparation [3], and food and cosmetics [4], the research on compound droplets continues to draw significant attention. Stone et al. [5] used the boundary integral method with axisymmetric assumption to numerically study the core-shell compound droplet with large deformation in linear flow; they also studied its rupture in the flow field. Hua et al. [6] used a numerical model based on the immersed boundary method to study the effects of droplet radius, surface tension ratio, and internal droplet position on the deformation of compound droplets in shear flows in both two and three dimensions and calculated the inclinations of internal and external droplets. Xu et al. [7] studied the coalescence kinetics of immiscible droplets through experiments, measured the evolution of the liquid bridge, compared it with miscible droplets, and proposed a theoretical model to analyze the influence of immiscibility in droplet coalescence. Yang et al. [8] used the phase field model to numerically study the dynamics of axisymmetric compound liquid threads and found that a larger inner liquid radius would significantly delay the evolution of compound liquid threads. They also found that when the inner liquid line radius was larger, the middle compound liquid thread would not shrink into droplets, while larger viscosity and surface tension ratios could delay the evolution of compound liquid threads. Wöhrwag et al. [9] proposed a model to simulate ternary fluid systems by incorporating a new free energy formula into the entropic lattice Boltzmann method (LBM). This model allows us to simulate problems with large density ratios and large surface tension ratios covering partial and complete wetting states.

All the above studies focused on the motion of compound droplets without a solid boundary. The fluid-solid interaction during the spreading of compound droplets on solid walls is more complex, and the related numerical work is more challenging. As the solid wall may have different shapes, curvatures, and wettabilities, it is worth conducting a lot of research in this area. Zhang et al. [10] proposed a geometric wetting condition for simulating ternary fluid flow by using the weighted contact angle model; they verified this model by simulating compound droplets on a substrate. Zhang et al. [11] used a diffuse interface method to simulate the morphology transformation of compound droplets on a straight wall, determined the irreversible and reversible configuration transformations, and verified the final equilibrium morphology of the droplets. Shi and Wang [12] proposed a phase field model for the dynamics of three-component immiscible fluids on a solid wall, used it to simulate the compound drop dynamics on a plane, and showed how the stability and deformation of the compound drop depend on the viscosity ratio of different fluids. In addition, they proposed an effective adaptive mesh refinement technique to improve the computation speed. Bhopalam et al. [13] combined a phase-field model using the ternary Navier–Stokes–Cahn–Hilliard equations with a neo-Hookean model for the solid, and studied the static wetting of a Janus compound droplet on a soft solid. They also simulated the interesting capillary origami of different compound droplets and the configuration shift of compound droplets on a soft solid under different physical parameters. Yang et al. [14] developed a novel diffuse-interface model to describe compound droplets in contact with solid. The wetting behavior of compound droplet on flat, inclined, spherical, and rough substrates was studied to verify the accuracy of their model. Huang [15] developed a hybrid lattice-Boltzmann finite-difference method for the simulation of ternary fluids near various immersed solid objects. Li et al. [16] proposed a numerical method for simulating three-phase flow with moving contact lines on complex surfaces within the framework of the color-gradient LBM. They validated their method through the simulations of a Janus droplet resting on a flat surface, a Janus droplet deposited on a cylinder, and the capillary intrusion of ternary fluids for various viscosity ratios. It was then used to study the dynamics of a compound droplet passing through an array of cylinders subject to a uniform incoming flow. Zhang and Huang [17] used the numerical method proposed in [15] to explore the wetting and spreading behavior of compound droplets on a two-dimensional wedge. Chowdhury et al. [18] studied the dynamics of capturing bubbles at a liquid-liquid interface under axisymmetric conditions by using the ternary Navier–Stokes–Cahn–Hilliard equations. They used a cone with varying wettability and a certain cone angle to remove the bubble, and then performed detailed numerical analyses of bubble detachment for a wide range of flow configurations.

There are many studies on three-phase flows, but there are still some insufficiencies in the existing works. For example, in Refs. [10,11,12], the solid walls involved are all planar, and the wetting of compound droplets on curved solid walls was not studied. Ref. [13] considered the challenging fluid-solid interactions, but it only studied two-dimensional problems. The test cases in Ref. [14] were mostly two-dimensional (only one was three-dimensional). Ref. [15] adopted the phase field theory based on the Cahn–Hilliard equation, and used the finite difference method to solve the phase field equations; it could not handle problems with large density ratios. Refs. [16,17] only studied problems with small to intermediate density ratios in two dimensions. Unlike most previous works, the numerical method in this paper is an axisymmetric LBM based on the conservative Allen–Cahn equations (CACEs) and it can simulate more realistic ternary fluid problems with large density ratios under axisymmetric conditions. It greatly saves computational resources when compared with full three-dimensional simulations. In addition, one major focus of this study is on the influence of curved solid wall properties (including the shape, curvature, and contact angle) on the wetting behavior of compound droplets, which has been rarely investigated in depth in the literature. This study was not only driven by curiosity, but also motivated by the following considerations. First, the knowledge on such wetting behavior may be helpful to understand the interaction between compound droplets and micro solid particles of various shapes. Second, the topological changes of compound droplets during such processes might be useful in some industrial processes aiming to separate different fluid components. Third, the diverse equilibrium morphologies of compound droplets on different curved surfaces could be employed to fabricate certain small components with special shapes (like microlens) in future.

In the remainder of this paper, Section 2 gives the numerical method used in the simulations, Section 3 presents some verifications of the method and the analyses on the results of compound droplets spreading on various curved walls, and Section 4 concludes this paper.

## 2. Numerical Method

The numerical method includes two parts: (1) the lattice Boltzmann equations (LBEs) for the axisymmetric CACEs used for the interface dynamics of a ternary fluid system, and (2) the LBEs for the axisymmetric incompressible NSEs to simulate the hydrodynamics with interfacial tension effects. The two parts are coupled with each other through the velocity, the physical properties of the fluids (i.e., density and viscosity), and the interfacial tension force. The two components are briefly introduced as follows.

### 2.1. Axisymmetric LBEs for the CACEs

In the conservative phase-field formulation for ternary fluids, the volume fraction (order parameter) $c_{i}\;(i=1,2,3)$ is used to identify fluid i and $c_{i}$ and satisfies $0\leq c_{i}\leq 1$ and the relation $\sum_{i=1}^{3}c_{i}=1$. At a given point, $c_{i}=1$ means that it is completely occupied by fluid i and $c_{i}=0$ means that fluid i is totally absent. There exist transition regions with $0<c_{i}<1$ that contain partially fluid i and also one or two of the other fluids. The interface is represented by the contour at $c_{i}=0.5$. Because $\sum_{i=1}^{3}c_{i}=1$, only two volume fractions are independent. Without loss of generality, we choose $c_{1}$ and $c_{2}$ here.

The CACEs for $c_{i}$ (i = 1, 2) with convection included are given by [19],

(1) $$\partial_{t}c_{i}+\vec{u}\cdot \nabla c_{i}=\nabla \cdot [m_{0}(\nabla c_{i}-(\lambda_{i}{\vec{n}}_{i}-{\vec{\beta }}_{i}))],$$

where $\vec{u}$ is the fluid velocity, $m_{0}$ is the (constant) mobility, $\lambda_{i}$ is defined as $4c_{i}(1-c_{i})/W$ with W being the interface thickness, ${\vec{n}}_{i}=\nabla c_{i}/(\left|\nabla c_{i}\right|+10^{-12})$ ($10^{-12}$ is added to avoid division-by-zero) is the unit normal vector of the interface of fluid i, and ${\vec{\beta }}_{i}$ is a Lagrange multiplier to enforce the condition $\sum_{i=1}^{3}c_{i}=1$, ${\vec{\beta }}_{i}=c_{i}\sum_{j=1}^{3}\lambda_{j}{\vec{n}}_{j}$. As for incompressible fluids where the velocity field is divergence free, $\nabla \cdot \vec{u}=0$, one can rewrite the convective term as $\vec{u}\cdot \nabla c_{i}=\nabla {\cdot (\vec{u}c}_{i})$. In Cartesian coordinates, the CACE for $c_{i}$ may be written as

(2) $$\partial_{t}c_{i}+\partial_{\alpha }(u_{\alpha }c_{i})=\partial_{\alpha }(m_{0}\partial_{\alpha }c_{i})-m_{0}\partial_{\alpha }(\lambda_{i}n_{i,\alpha }-\beta_{i,\alpha }),$$

and in cylindrical coordinates for axisymmetric problems, it reads [19],

(3) $$\partial_{t}c_{i}+\partial_{\alpha }(u_{\alpha }c_{i})+\frac{1}{r}c_{i}u_{r}=\partial_{\alpha }(m_{0}\partial_{\alpha }c_{i})+\frac{m_{0}}{r}[\partial_{r}c_{i}-(\lambda_{i}n_{i,r}-\beta_{i,r})]-m_{0}\partial_{\alpha }(\lambda_{i}n_{i,\alpha }-\beta_{i,\alpha }),$$

where r is the coordinate in the radial direction (the other coordinate z is in the axial direction). Note that Equation (3) can be rewritten in conservative form as,

(4) $$\partial_{t}(rc_{i})+\partial_{\alpha }(ru_{\alpha }c_{i}+m_{0}c_{i}\delta_{\alpha r})=\partial_{\alpha }[{m_{0}\partial }_{r}(rc_{i})-m_{0}r(\lambda_{i}n_{i,\alpha }-\beta_{i,\alpha })].$$

We first present the axisymmetric LBEs using the multiple-relaxation-time (MRT) collision model [20] to solve the axisymmetric CACEs. Two sets of distribution functions (DFs), $g_{l}$ and $h_{l}$, are used for the volume fractions $c_{1}$ and $c_{2}$, respectively. Here we only give the details on $g_{l}$ for $c_{1}$ (the formulations on $h_{l}$ for $c_{2}$ are similar and omitted for brevity). Inspired by [19,20], we adopt the following MRT version of the LBEs on $g_{l}$ for the axisymmetric CACE for $c_{1}$,

(5) $$g_{l}(\vec{x}+{\vec{e}}_{l}\delta_{t},t+\delta_{t})-g_{l}(\vec{x},t)={-(M^{-1}S_{g}M)}_{lm}[g_{m}(\vec{x},t)-g_{m}^{eq}(\vec{x},t)]+\delta_{t}G_{l}(\vec{x},t),$$

where ${\vec{e}}_{l}$ is the lattice velocity along the direction l (l=0,1,⋯, 8 for the D2Q9 velocity model adopted here), $\delta_{t}$ is the time step, and M is a matrix that transforms the vector of DFs into a vector of moments [21]. The volume fraction $c_{1}$ is obtained from the DFs $g_{l}$ as

(6) $$c_{1}=\frac{1}{r}\sum_{l}g_{l}$$

For brevity, the details of the MRT collision model, the boundary conditions near the solid wall, and the calculation of the spatial derivatives are given in Appendix A.

### 2.2. Axisymmetric LBEs for the NSEs

The axisymmetric LBEs for the NSEs are quite similar to those in [22] except for a few changes related to the extension from binary to ternary fluids. For completeness, it is briefly introduced as follows. In LBM, the incompressible NSEs for ternary fluid flows with variable fluid properties are usually approximated by [23],

(7) $$\frac{\partial p}{\partial t}+\rho (c_{1},c_{2})c_{s}^{2}\nabla \cdot \vec{u}=0,$$

(8) $$\rho (c_{1},c_{2})(\frac{\partial \vec{u}}{\partial t}+\vec{u}\cdot \nabla \vec{u})=-\nabla p+{\vec{F}}_{ST}+\nabla \cdot [\eta (c_{1},c_{2})(\nabla \vec{u}+{\left(\nabla \vec{u}\right)}^{T})],$$

where p is the hydrodynamic pressure, $\rho (c_{1},c_{2})$ is the density linearly interpolated from the volume fractions $c_{1}$ and $c_{2}$ as $\rho \left(c_{1},c_{2}\right)=\rho_{1}c_{1}+\rho_{2}c_{2}+\rho_{3}\left(1-c_{1}-c_{2}\right)$($\rho_{i}$ is the density of the i-th fluid), and $\eta (c_{1},c_{2})=\rho (c_{1},c_{2})\upsilon (c_{1},c_{2})$ is the dynamic viscosity with $\upsilon (c_{1},c_{2})$ being the kinematic viscosity found from those of fluid i(i=1,2,3) as ${\left[\upsilon (c_{1},c_{2})\right]}^{-1}=c_{1}\upsilon_{1}^{-1}+c_{2}\upsilon_{2}^{-1}+(1-c_{1}-c_{2})\upsilon_{3}^{-1}$ [23]. The dynamic viscosity of fluid i is $\eta_{i}=\rho_{i}\upsilon_{i}$, and ${\vec{F}}_{ST}$ is the interfacial tension force; its expression is given in Appendix A.

In Cartesian coordinates, the LBEs using the single-relaxation-time (SRT) collision model to recover Equations (7) and (8) read [24],

(9) $$f_{l}\left(\vec{x}+{\vec{e}}_{l}\delta_{l},t+\delta_{l}\right)-f_{l}(\vec{x},t)=-\frac{1}{\tau_{f}}(f_{l}-f_{l}^{eq})+(1-\frac{1}{2\tau_{f}})({\vec{e}}_{l}-\vec{u})\cdot [\nabla \rho c_{s}^{2}(\Gamma_{l}-\Gamma_{l}(0))+{\vec{F}}_{ST}\Gamma_{l}]\delta_{t},$$

where $f_{l}$ and $f_{l}^{eq}$ are the DFs and equilibrium DFs for the hydrodynamic variables. $f_{l}^{eq}$ is given by,

(10) $$f_{l}^{eq}=w_{l}[p+\rho c_{s}^{2}(\frac{1}{c_{s}^{2}}e_{l\alpha }u_{\alpha }+\frac{1}{2c_{s}^{4}}(e_{l\alpha }e_{l\beta }-c_{s}^{2}\delta_{\alpha \beta })u_{\alpha }u_{\beta })],$$

and the relaxation parameter $\tau_{f}$ is related to the kinematic viscosity as $\upsilon =c_{s}^{2}(\tau_{f}-0.5)\delta_{t}$. $\Gamma_{l}$ is the dimensionless DFs given by: $\Gamma_{l}=w_{l}(1+\frac{1}{c_{s}^{2}}e_{l\alpha }u_{\alpha }+\frac{1}{2c_{s}^{4}}e_{l\alpha }u_{\alpha }e_{l\beta }u_{\beta }-\frac{1}{2c_{s}^{2}}u_{\alpha }u_{\alpha })$. The fluid pressure and momentum are calculated as,

(11) $$p=\sum_{l}f_{l}+\frac{1}{2}\delta_{t}(\vec{u}\cdot \nabla \rho c_{s}^{2}),$$

(12) $$\rho \vec{u}=\frac{1}{c_{s}^{2}}\sum_{l}f_{l}{\vec{e}}_{l}+\frac{1}{2}\delta_{t}{\vec{F}}_{ST},$$

To simulate axisymmetric three-phase flows, the equations must be properly changed to account for axisymmetric effects. Specifically, the axisymmetric formulation used here is modified from that in [22]. When azimuthal flows are absent, the target axisymmetric NSEs to be recovered by the axisymmetric LBEs are

(13) $$\frac{1}{c_{s}^{2}}\frac{\partial p}{\partial t}+\frac{\partial }{\partial_{xa}}(\rho u_{a})=-\frac{\rho u_{r}}{r},$$

(14) $$\rho (\frac{\partial u_{\alpha }}{\partial t}+u_{\beta }\frac{\partial u_{\alpha }}{\partial x_{\beta }})=-\frac{\partial p}{\partial x_{\alpha }}+F_{ST,,\alpha }+\frac{\partial }{\partial x_{\beta }}[\eta (\frac{\partial u_{\alpha }}{\partial x_{\beta }}+\frac{\partial u_{\beta }}{\partial x_{\alpha }})]+F_{axisym,\alpha }-\frac{1}{r}\rho u_{r}u_{\alpha },$$

where the additional force due to axisymmetric effects $F_{axisym,\alpha }$ is given by,

(15) $$F_{axisym,\alpha }=\frac{\eta }{r}(\frac{\partial u_{\alpha }}{\partial r}+\frac{\partial u_{r}}{\partial x_{\alpha }}),$$

As compared with the expression in [25], Equation (14) is more simplified because a different form of interfacial tension force is used here.

The axisymmetric LBEs for Equations (13) and (14) read [25],

(16) $$f_{l}\left(\vec{x}+{\vec{e}}_{l}\delta_{t},t+\delta_{t}\right)-f_{l}(\vec{x},t)=-\frac{1}{\tau_{f}}(f_{l}-f_{l}^{eq})+(1-\frac{1}{2\tau_{f}})[S_{l}+S_{l}^{\prime }+S_{l}^{\prime \prime }]\delta_{t},$$

where $S_{l}=(\vec{e_{l}}-\vec{u})\cdot [\nabla \rho c_{s}^{2}(\Gamma_{l}-\Gamma_{l}(0))+{\vec{F}}_{ST}\Gamma_{l}]$ is a source term having the same form as in Equation (9), $S_{l}^{\prime }$ and $S_{l}^{\prime \prime }$ are another two source terms added due to the axisymmetric effects [25],

(17) $$S_{l}^{\prime }=w_{l}c_{s}^{2}(-\frac{\rho u_{r}}{r}),\;S_{l}^{\prime \prime }=({\vec{e}}_{l}-\vec{u})\cdot ({\vec{F}}_{axisym}-\frac{\rho u_{r}\vec{u}}{r})\Gamma_{l},$$

The fluid pressure and momentum in the axisymmetric formulation are computed from

(18) $$p=\sum_{l}f_{l}+\frac{1}{2}\delta_{t}(\vec{u}\cdot \nabla \rho c_{s}^{2}-\rho c_{s}^{2}\frac{u_{r}}{r}),$$

(19) $$\rho \vec{u}=\frac{1}{c_{s}^{2}}\sum_{l}f_{l}{\vec{e}}_{l}+\frac{1}{2}\delta_{t}({\vec{F}}_{ST}+{\vec{F}}_{axisym}-\frac{\rho u_{r}\vec{u}}{r}),$$

It is noted that the new pressure and velocity are computed in an iterative manner as the above two equations are coupled with each other. The velocity gradient terms in Equation (15) are evaluated by the 2nd-order central difference schemes. For conciseness, the MRT model [21] and the Lax–Wendroff streaming scheme used to suppress the chequerboard effect are described in detail in Appendix A.

## 3. Results and Discussions

This work mainly studies the wetting and spreading of compound droplets on different types of solid walls and focuses on whether droplet separation occurs during the wetting process. Commonly, compound droplets may take two different configurations in the absence of solid walls, namely, the core-shell type and the Janus type. In this work, we concentrate exclusively on Janus droplets. For small droplets with relatively large interfacial tension, the effect of gravity can be ignored. In this section, we first present the calculation of some important quantities and the physical model, and then verify our numerical method by checking the shape of compound droplets on different walls in equilibrium state. After that, we will discuss the influence of several factors on the droplet separation phenomenon, including the wall type, curvature, contact angle, the interfacial angles, and the density ratio.

In phase field simulations of ternary fluid flows, the free energy functional F is defined by [26]

(20) $$F(c_{1},c_{2},c_{3},\nabla c_{1},\nabla c_{2},\nabla c_{3})=\int_{V}(\frac{12}{W}F_{0}(c_{1},c_{2},c_{3})+\frac{3}{4}W\gamma_{1}{\left|\nabla c_{1}\right|}^{2}+\frac{3}{4}W\gamma_{2}{\left|\nabla c_{2}\right|}^{2}+\frac{3}{4}W\gamma_{3}{\left|\nabla c_{3}\right|}^{2})dV,$$

where the bulk free energy density $F_{0}$ is given by

(21) $$F_{0}(c_{1},c_{2},c_{3})=\gamma_{1}c_{1}^{2}{\left(1-c_{1}\right)}^{2}+\gamma_{2}{c_{2}^{2}(1-c_{2})}^{2}+\gamma_{3}c_{3}^{2}{\left(1-c_{3}\right)}^{2},$$

and the latter three terms in Equation (20) represent the interfacial energies associated with $c_{i}(i=1,\;2,\;3)$. Note that an additional term in [26] for total spreading cases is omitted because here we only consider partial spreading cases. With Equation (21), one may separate F into three parts as $F=\sum_{i=1}^{3}F_{i}$ where $F_{i}$ represents the contribution related to $c_{i}$,

(22) $$F_{i}=\int_{V}(\frac{12}{W}\gamma_{i}c_{i}^{2}{\left(1-c_{i}\right)}^{2}+\frac{3}{4}W\gamma_{i}{\left|\nabla c_{i}\right|}^{2})dV=\frac{3\gamma_{i}}{4W}\int_{V}(16c_{i}^{2}{\left(1-c_{i}\right)}^{2}+W^{2}{\left|\nabla c_{i}\right|}^{2})dV,$$

In the conservative phase field framework, the free energies mostly come from the interfacial regions because they almost vanish in the bulk regions where $c_{i}\approx 0$ or $c_{i}\approx 1$, $\left|\nabla c_{i}\right|\approx 0$. In actual simulations,$F_{i}$ is easily obtained from the fields for the volume fraction $c_{i}$ and its gradient. However, for specific problems, one is usually more interested in the interfacial energies associated with an interface, e.g., $E_{s,ij}$ between fluids i and j (than in $F_{i}$). Fortunately, $E_{ij}$ can be found from $F_{i}$ as

(23) $$E_{s,ij}=(\gamma_{i}+\gamma_{j})(\frac{F_{i}}{\gamma_{i}}+\frac{F_{j}}{\gamma_{j}}-\frac{F_{k}}{\gamma_{k}}),$$

where i,j, and k are different. The total kinetic energy and the kinetic energy of fluid i may be calculated as

(24) $$E_{k,t}=\int_{V}\frac{1}{2}\rho (c_{1},c_{2}){\left|\vec{u}\right|}^{2}dV,$$

(25) $$E_{k,i}=\int_{V}N(c_{i})\frac{1}{2}\rho_{i}{\left|\vec{u}\right|}^{2}dV,$$

where $N\;(c_{i})=1$ for $\;c_{i}>0.5$ and $N\;(c_{i})=0$ otherwise. Note that in cylindrical coordinates for axisymmetric problems, $\int_{V}()dV=\int_{V}()(2\pi r)dzdr$.

### 3.1. Physical Model and Parameters

**Figure 1:**
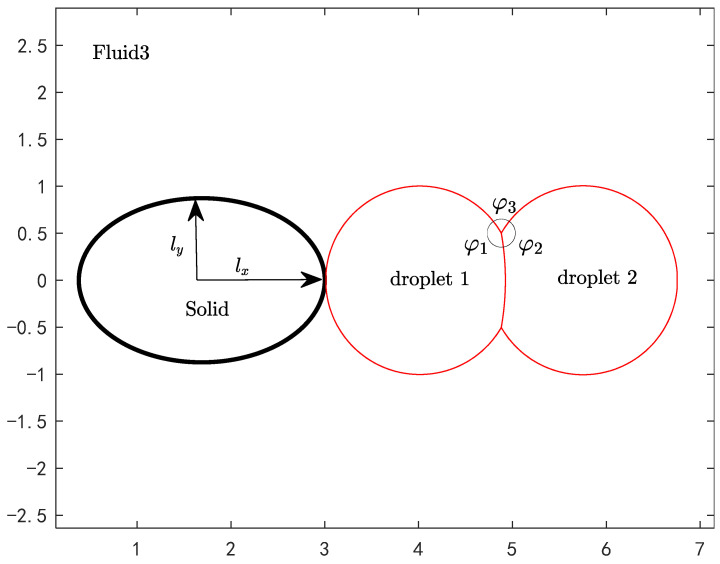
Physical model diagram.

Several kinds of solid walls with different geometries will be considered. To illustrate the basic physical settings, we choose the wetting of compound droplets on an ellipsoidal wall (corresponding to a prolate spheroid in three dimensions) as an example. As shown in Figure 1, the initial compound droplet of the Janus type is composed of two droplets of different components (fluid 1 on the left and fluid 2 on the right) that have been fused together to be in static equilibrium. The volumes of the two droplets are both equal to 4.189 (about the volume of a sphere with a radius of $R_{0}=1$). In other words, we chose the reference length as $L_{r}=R_{0}$. Note that the initial state may be also realized by placing two spherical droplets of radius 1, one of fluid 1 and the other of fluid 2, in contact and letting the system evolve freely under the action of interfacial tension (with dissipation) to equilibrium. To circumvent this step, we employed the analytical solution for the final equilibrium configuration under given interfacial angles. The surrounding environment is fluid 3. The angles $\varphi_{i}(i=1,\;2,\;3)$ at the triple junction measured in fluid i in Figure 1 are called interfacial angles and, for cases other than total spreading, they satisfy

(26) $$\sum_{i=1}^{3}\varphi_{i}=2\pi ,\;sin\varphi_{3}/\sigma_{12}=sin\varphi_{2}/\sigma_{13}=sin\varphi_{1}/\sigma_{23}.$$

The domain used in our simulations is a rectangle with a size of $L_{x}\times L_{y}.$ The semi-axis lengths in the x-direction, y-direction, and z-direction of the prolate spheroid are $l_{x}=1.310$, $l_{y}=l_{z}=0.8736$ (in order to meet the axisymmetric requirement, it is necessary to have $l_{y}=l_{z}$), and its volume of the ellipsoid is $V=4\pi l_{x}l_{y}l_{z}/3=4.189$ (the same as the volume of a unit sphere). For conciseness, in the following only $l_{y}$ is given for a prolate or oblate spheroid and $l_{z}=l_{y}$ will be omitted. Note that the x- and y-axes in Figure 1 correspond to the z- and r-axes in the cylindrical coordinate system, respectively. Associated with the three kinds of interfaces in a ternary fluid system, there are three different contact angles (CAs) on a wall and these CAs are not independent. From the force balance of a compound droplet on a wall in equilibrium, they must satisfy the following condition [15]

(27) $${sin\varphi }_{2}cos\theta_{13}-{sin\varphi }_{3}cos\theta_{12}-{sin\varphi }_{1}cos\theta_{23}=0.$$

where $\theta_{ij}$ represents the contact angle for the interface between fluids i and j on the wall measured in fluid i ($\theta_{ij}+\theta_{ji}=\pi$). Periodic boundary conditions are used on the left and right sides of the domain. Solid wall boundary conditions are used on the top side, and symmetrical boundary conditions are used on the bottom side. For simplicity, the density ratio and the kinematic viscosity ratio of fluid 1 to fluid 2 are fixed at $r_{\rho 12}=1$ and $r_{\upsilon 12}=1$ (note that the present method can handle other values of $r_{\rho 12}$ and $r_{\upsilon 12}$). The density ratio and the kinematic viscosity ratio of fluid 1 to fluid 3 are ${\;r}_{\rho 13}$ and $r_{\upsilon 13}$, respectively.

It is noted that the present setting in Figure 1 is similar to “Configuration L (lens)” in [11], which also considered axisymmetric problems. However, they mainly focused on the equilibrium states of compound droplets on a planar wall at some fixed interfacial angles. In contrast, we will study the dynamic wetting processes on different curved walls at a variety of conditions, during which the compound droplet may undergo significant topological changes. In addition, [13,16] also considered a Janus droplet near a wall. However, the arrangement of the droplets with respect to the wall is different from the present one. In their studies, both constituent droplets wetted the wall. The problem studied by them was two dimensional and does not actually exist in reality.

The reference velocity is chosen as $U_{r}=\sigma_{12}/\eta_{1}$, which is derived from the interfacial tension $\sigma_{12}$ and the dynamic viscosity of fluid 1 $\eta_{1}(=\rho_{1}\nu_{1})$, and the reference time is derived from $L_{r}$ and $U_{r}$ as $T_{r}=\frac{L_{r}}{U_{r}}=\frac{L_{r}\eta_{1}}{\sigma_{12}}$. All length and time quantities are measured in $L_{r}$ and $T_{r}$, respectively. From these, one can calculate the Reynolds number as $Re=\frac{\rho_{r}U_{r}L_{r}}{\eta_{r}}=\frac{\rho_{1}\sigma_{12}L_{r}}{\eta_{1}^{2}}$ and the Weber number as $We=\frac{\rho_{r}U_{r}^{2}L_{r}}{\sigma_{12}}=Re$. The capillary number is found to be $Ca=\frac{\eta_{r}U_{r}}{\sigma_{12}}=\frac{We}{Re}=1$ and the Ohnesorge number is $Oh=\frac{\eta_{1}}{\sqrt{\rho_{1}\sigma_{12}L_{r}}}=\frac{1}{\sqrt{Re}}$. In the simulations, $L_{r}$ was discretized by $N_{L}$ grids and $T_{r}$ was discretized by $N_{t}$ time steps. Then, the grid size and time step are obtained as: $\delta_{x}=L_{r}/N_{L}$, $\delta_{t}=T_{r}/N_{t}$. In phase-field simulations, the interface thickness and mobility must be properly set. Regarding the former, $W/\delta_{x}=$ 5 was used as in many phase-field simulations in the literature and $N_{L}$ was set to be larger enough to make the Cahn number $C_{n}=W/L_{r}=(W/\delta_{x})/N_{L}$ small. For the latter, we used the mobility $m_{0}=0.1$ (in lattice units) empirically (which provides reasonable results under most circumstances). In order to ensure the accuracy of the simulation, tests on the grid density and domain size were carried out for a typical case (see Appendix B and Appendix C for details). Based on the grid density tests, to balance the requirements of accuracy and computational overhead, $N_{L}=50$ was used in the following.

### 3.2. Numerical Validation

**Figure 2:**
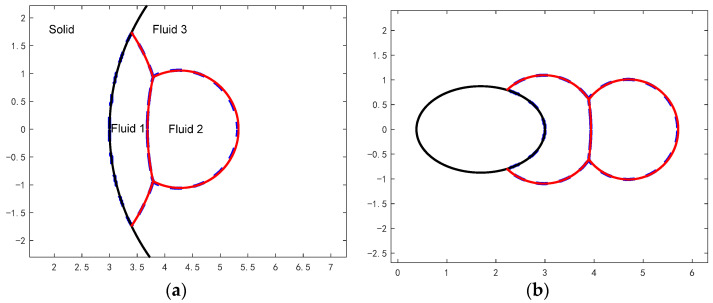

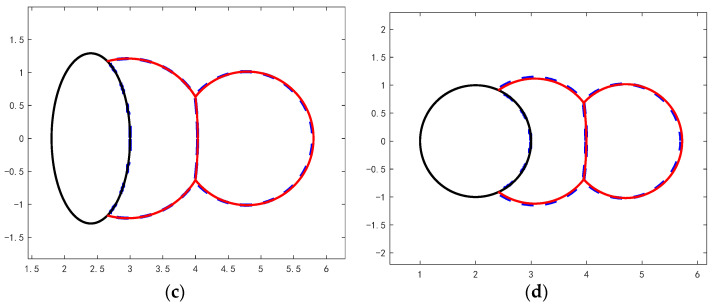
Verification of equilibrium shapes of compound droplets on different walls. The black solid lines represent the solid walls, the blue dashed lines are the interfaces obtained by our simulations, and the red solid lines are from the corresponding analytical solutions. The contact angle on the wall (for fluids 1 and 3) is $\theta_{13}=60{}^{\circ}$. The interfacial angles are (**a**,**d**): ${\;\varphi }_{1}=150{}^{\circ}$, $\varphi_{2}=130{}^{\circ}$, $\varphi_{3}=80{}^{\circ}$; (**b**,**c**): ${\;\varphi }_{1}=155{}^{\circ}$, $\varphi_{2}=135{}^{\circ}$, $\varphi_{3}=70{}^{\circ}$.

First, the equilibrium shapes of compound droplets on several types of solid walls are calculated by numerical simulations and compared with the corresponding analytical solutions based on the equilibrium conditions (see Appendix D and Appendix E for the derivation of the analytical solutions of the equilibrium state of the compound droplet on different walls). The total simulation time in each case is t=600. At this time, the total kinetic energy of the compound droplet has approached zero. The comparisons between the numerical and analytical results are shown in Figure 2, where the blue dashed lines are the interfaces obtained by our simulations and the red solid lines are from the corresponding analytical solutions. The common parameters used in the simulations are: $\theta_{13}=60{}^{\circ}$, $r_{\rho 12}=1$, $r_{\rho 13}=825$, $r_{\upsilon 12}=1$, $r_{\upsilon 13}=0.06$, Re=400 (Oh=0.05). Note that the other two CAs in Equation (27) are not important here because only the left droplet wets the wall. The interfacial angles of Figure 2a,d are:${\;\varphi }_{1}=150{}^{\circ}$, $\varphi_{2}=130{}^{\circ}$, $\varphi_{3}=80{}^{\circ}$. Interfacial angles of Figure 2b,c are:${\;\varphi }_{1}=155{}^{\circ}$, $\varphi_{2}=135{}^{\circ}$, $\varphi_{3}=70{}^{\circ}$. The solid wall in Figure 2a is concave (i.e., having negative curvature) and its radius of curvature is $R_{c}=4$. In other words, the droplet wets the inner surface of a sphere. The solid walls in Figure 2b–d are all convex (i.e., the droplet wets the outer surface of an ellipsoid or a sphere). In Figure 2b the prolate spheroid has semi-axis lengths in the x-direction and y-direction $l_{x}=1.310$, $l_{y}=0.8736$. In Figure 2c, the oblate spheroid has semi-axis lengths $l_{x}=0.6$, $l_{y}=1.291$. In Figure 2d, the sphere has a radius of R=1. From Figure 2, it is observed that the equilibrium interface positions by our simulation are close to the theoretical solutions for all cases. Thus, our numerical method should be reliable to predict the wetting characteristics of Janus droplets on curved walls. It can also be seen from Figure 2 that as the curvature of solid wall varies, the shape of droplet 2 changes slightly whereas the shape of droplet 1 changes more significantly. When the wall curvature decreases with all other conditions fixed, the contact area between droplet 1 and the solid wall increases as found through the comparison between Figure 2a,d (or through the comparison between Figure 2b,c).

### 3.3. Wetting of Compound Droplets on Three Types of Solid Walls

**Figure 3:**
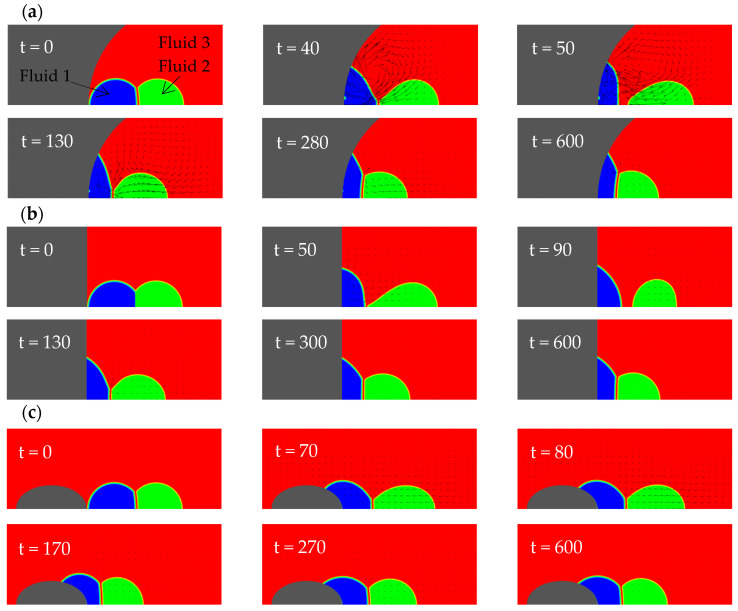
Flow field evolutions of compound droplets wetting a solid wall with different shapes: (**a**) a concave wall with a radius of curvature $R_{c}=4$; (**b**) a planar wall; (**c**) a prolate spheroid with $l_{x}=1.310$, $l_{y}=0.8736$.

In this section, three types of solid walls are considered: a concave spherical surface with a radius of curvature $R_{c}=4$, a plane, and a prolate spheroid with the semi-axis lengths $l_{x}=1.310$, $l_{y}=0.8736.$ The purpose is to explore the effect of wall shape on the dynamic behavior of a Janus droplet during its spreading on the wall. Figure 3 shows the wetting processes on the three kinds of walls. The common parameters used in the simulations are:${\;\varphi }_{1}=150{}^{\circ}$, $\varphi_{2}=130{}^{\circ}$, $\varphi_{3}=80{}^{\circ}$, $\theta_{13}=60{}^{\circ}$, $r_{\rho 12}=1$, $r_{\rho 13}=825$, $r_{\upsilon 12}=1$, $r_{\upsilon 13}=0.06$, Re=400 (Oh=0.05). It is noted that the Reynolds number and the physical properties of the fluids (including the density and viscosity ratios) in this section are also used in all subsequent sections (unless specified otherwise).

It can be seen from Figure 3 that the motions of the compound droplet for all cases follow a similar sequence. First, the left droplet spreads on the wall. This initiates a capillary wave propagating from the contact line along the interface between fluids 1 and 3. After some time, the wave reaches the three-phase point (where the three fluids meet) and continues in two directions: one along the interface between the two drops (i.e., fluids 1 and 2) and the other along that between fluids 2 and 3. During its propagation, the capillary wave is damped to some extent due to viscous dissipation. It also takes some time for it to arrive at the three-phase point and subsequently affect the right droplet. As the right droplet has no direct contact with the wall, it is only affected by the interfacial tension of the interface between the two droplets (for convenience, called “fusion interface” below). Thus, its deformation and motion are lagging the left. When the intrinsic contact angle is the same, the concave wall “bends” the interface between fluid 1 and 3 more heavily than the planar and convex walls because the initial shape of the left droplet deviates from its (imagined) equilibrium configuration on the concave wall most severely (in other words, for the concave wall case the system initially has the largest interfacial energy potential). This causes the left droplet to spread the fastest and deform the most violently on the concave wall. During this process, the left droplet disconnects with the right droplet the earliest (see the snapshot at t=50 in Figure 3a). For the planar wall case, the initial potential to drive the left droplet is not as large as the concave wall case but is still enough to split the Janus droplet at a later time (see the snapshot at t=90 in Figure 3b). For the convex wall, the driving potential is the smallest and is insufficient to split the compound droplet (as see in Figure 3c). From Figure 3, it is also observed that, in the end, the two droplets remain in the form of a compound droplet for all three cases. On both the concave and planar walls, after the separation occurs, the left droplet slows down whereas the right droplet keeps moving towards the left because it was accelerated to obtain a certain momentum by the interfacial tension force from the left droplet. After some time, the right droplet touches the left one again and they merge to form a different Janus droplet on the wall that eventually reaches static equilibrium. Thus, the observed splitting of Janus droplet is only transient. Making use of this splitting stage to permanently separate the two fluids is beyond the scope of this work. Here we simply focus on the events of topological changes during the wetting process, which are of interest on their own.

Here it is helpful to examine the changes in the energy of the whole system (including the wall) between the initial state and the final equilibrium state for the three cases. In these two states, the velocity is zero everywhere, and therefore the system energy only consists of the interfacial energies. Denote the total area of the solid surface as $A_{s}$ and the area of the surface wetted by the left droplet (of fluid 1) as $A_{w}$. The area of the interface between fluids i and j is $A_{ij}$. The interfacial tension between the solid wall and fluid i is $\sigma_{is}$. In the initial state (labelled with a superscript ${\;}^{0}$), the system energy may be expressed as $E_{t}^{0}=\sigma_{12}A_{12}^{0}+\sigma_{13}A_{13}^{0}+\sigma_{23}A_{23}^{0}+\sigma_{3s}A_{s}$. In the final state (labelled with a superscript ${\;}^{f}$), it becomes $E_{t}^{f}=\sigma_{12}A_{12}^{f}+\sigma_{13}A_{13}^{f}+\sigma_{23}A_{23}^{f}+\sigma_{1s}A_{w}+\sigma_{3s}{(A}_{s}-A_{w})$. Thus, the energy change from the initial state to the final state is $\Delta E_{t}^{0\to f}=E_{t}^{0}-E_{t}^{f}=\sigma_{12}\left(A_{12}^{0}-A_{12}^{f}\right)+\sigma_{13}{(A}_{13}^{0}-A_{13}^{f})+\sigma_{23}{(A}_{23}^{0}-A_{23}^{f})+(\sigma_{3s}-\sigma_{1s})A_{w}$. From Young’s equation on the contact angle, one has $\sigma_{3s}=\sigma_{1s}+\sigma_{13}cos\theta_{13}$. Then, one has $∆E_{t}^{0\to f}=\sigma_{12}∆A_{12}+\sigma_{13}∆A_{13}+\sigma_{23}∆A_{23}+\sigma_{13}cos\theta_{13}A_{w}$ where $∆A_{ij}=A_{ij}^{0}-A_{ij}^{f}$. Without loss of generality, we consider the energy change scaled by the interfacial tension between fluids 1 and 2, $\Delta E_{t}^{0\to f}/\sigma_{12}$. The initial areas $A_{ij}^{0}$ are all given. The final areas $A_{ij}^{f}$ and the wetted area $A_{w}$ are obtained from the analytical solutions. In the end, the energy changes are found to be $∆E_{t}^{0\to f}/\sigma_{12}=2.887$ for the case on the concave wall, $∆E_{t}^{0\to f}/\sigma_{12}=2.864$ for the planar wall, and $∆E_{t}^{0\to f}/\sigma_{12}=1.792$ for the ellipsoidal wall. These results provide quantitative evidence for the above discussions on the driving potential.

**Figure 4:**
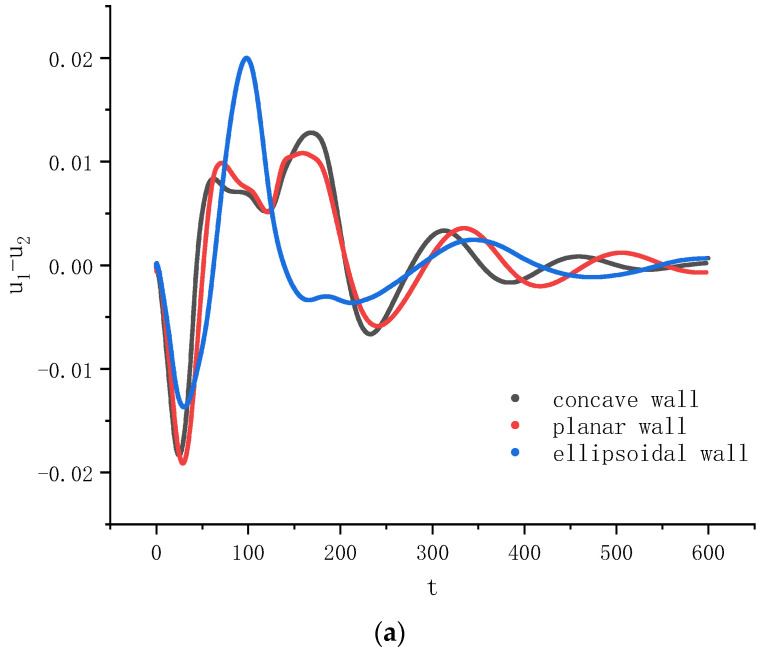

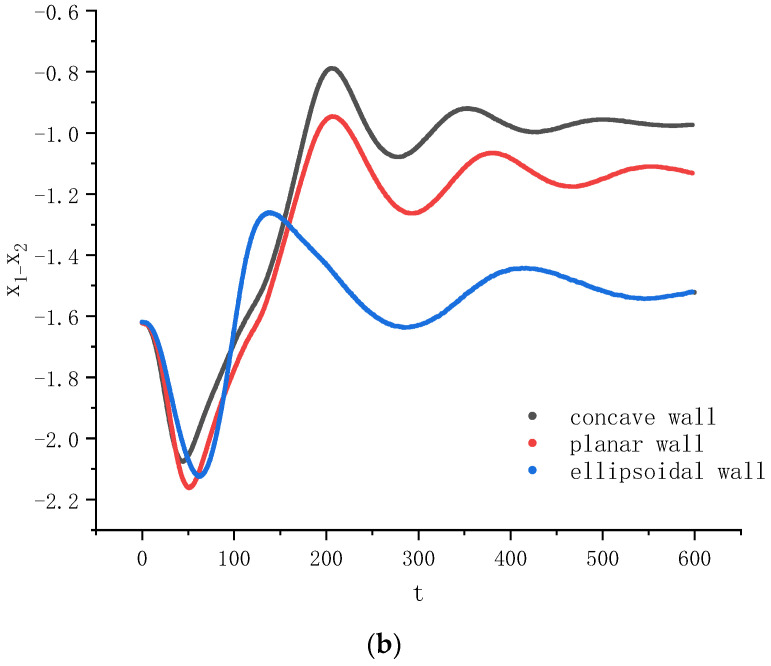
Evolutions of (**a**) the relative velocity and (**b**) the relative position along the axial direction between the two droplets’ centroids on the concave, planar and ellipsoidal walls.

For this problem, we monitored the centroid velocities and positions of the droplets along the x-direction $u_{i}$ and $x_{i}$ (i=1 for the left droplet and i=2 for the right droplet). Take the left droplet as an example. Its centroid velocity $u_{1}$ was calculated by,

(28) $$u_{1}=\frac{\int_{A\left|c_{1}>0.5\right.}yu(x,y)dxdy}{\int_{A\left|c_{1}>0.5\right.}ydxdy},$$

where $A\left|c_{1}>0.5\right.$ represents the region where $c_{1}>0.5$. Figure 4 shows the evolutions of the relative velocity and position between the left and right droplets’ centroids along the x-axis, $u_{1}-u_{2}$ and $x_{1}-x_{2}$, respectively, for the three cases in Figure 3. It can be seen from Figure 4a that in the early stage (t<80), the relative velocities are negative for all cases, and its magnitude first increases and then decreases. During this stage, the maximum magnitude of the relative velocity (abbreviated as MMRV for brevity below) for the third case (the ellipsoidal wall) is the largest, the MMRV for the planar wall is the smallest, and that for the concave wall is in between. Interestingly, although the MMRV is larger on the concave wall than that on the ellipsoidal wall, the maximum distance between the two droplets’ centroids is smaller on the ellipsoidal wall during the early stage, as seen from Figure 4b. This is because the change in distance is determined by the integration of the relatively velocity in time, not by the MMRV. It can also be seen in Figure 4b that the maximum distance is the largest for the flat wall. From Figure 3 and Figure 4, it seems that whether the Janus droplet splits is more related with the MMRV than with the maximum distance between the two droplets.

For convenience, in subsequent discussions we set the flat wall as a baseline. The concave wall extends towards the upper and right side (in regions not far away from the axis, predominantly towards the upper side for $R_{c}=4$) on the right of the baseline. This limits the leftwards motion of the left droplet to some extent. At the same time, the interfacial tension force near the contact line gives the most violent pull to the left droplet towards the upper and right side, somehow tearing the left droplet off after a certain time. As we consider axisymmetric problems here, the upper direction is the radial direction, and the contact line corresponds to a circle in three dimensions. When the contact line moves further away from the axis, the perimeter of the circle (along which the tugging force acts) increases (proportional to the radial coordinate). In contrast, the ellipsoidal wall extends towards the left and upper side. For the specific prolate spheroid considered here, the direction is predominantly towards the left. Therefore, the increase in the perimeter of the contact line during wetting is less significant than that in the first case. In addition, the tugging force on the contact line is smaller in the third case. The differences in these two factors partially explain why the Janus droplet splits in the first case but not in the third. Intuitively, one can imagine two extreme scenarios (corresponding to two types of mechanisms) for the separation to occur. The first is the “relative motion induced separation”, in which the left droplet is accelerated leftwards extremely fast and the right droplet almost stays in its original place due to inertia. The second is the “deformation induced separation”, in which the left droplet deforms (to become relatively flat) very quickly and breaks the connection with the right droplet. Of course, in reality, both mechanisms may play some role concurrently. For the above three cases, the second mechanism seems to be more effective than the first one; as seen in Figure 3, the left droplet experiences significant deformations on the flat and the concave walls and separation occurs in these two cases. The situation on the convex wall is the opposite.

**Figure 5:**
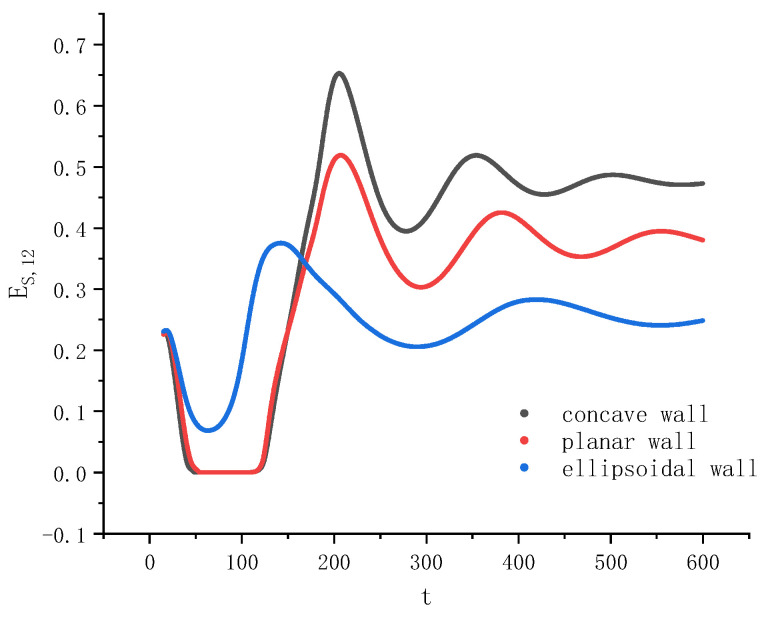
Evolution of the interfacial energy of the fusion interface between the two droplets with time during the wetting on three types of walls. Note that the values of the vertical coordinate have been multiplied by the Weber number (We=400).

Figure 5 shows the evolution of the interfacial energy of the fusion interface between the two droplets with time for the three cases on different walls. The expression of this interfacial energy is given by Equation (23) with i=1, j=2. Because, during the period of droplet separation, the fuse interface disappears and the interfacial energy $E_{s,12}$ becomes zero, it is straightforward to determine from Figure 5 when the compound droplet splits and at what time the left and right droplets contact each other again. As seen in Figure 5, initially the interfacial energy decreases the fastest on the concave wall and the slowest on the ellipsoid. The change of $E_{s,12}$ on the planar wall looks close to that on the concave wall in the initial stage, during which $E_{s,12}$ vanishes for a certain time. In contrast, $E_{s,12}$ always remains positive on the ellipsoid, indicating that droplet separation never occurs. These results are consistent with the previous findings. Through the comparison of the interfacial energy in the late stage when the system approaches equilibrium on the three kinds of solid walls, it can also be found that $E_{s,12}$ on the concave wall is the largest and that on the ellipsoid is the smallest.

**Figure 6:**
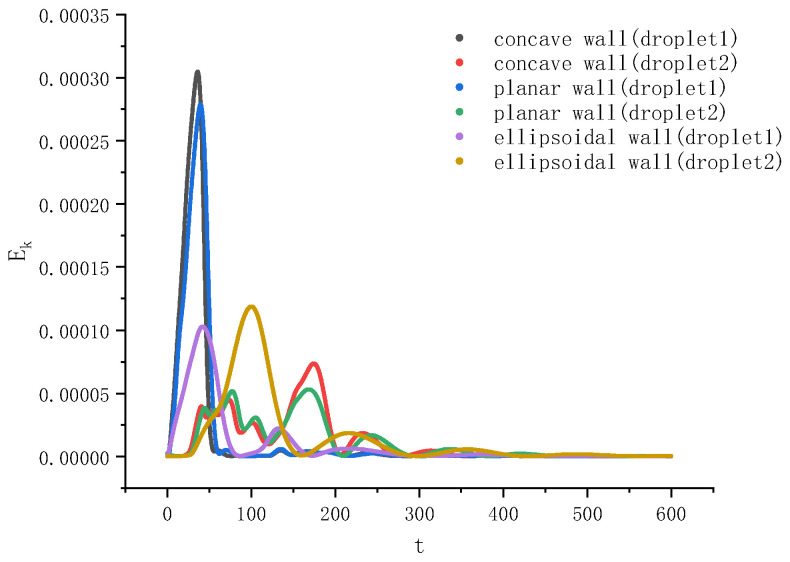
Evolution of the kinetic energy of the two droplets with time during wetting on three types of walls.

The evolution of the kinetic energy of the two droplets was also monitored, as shown in Figure 6. From this figure, it is found that the maximum kinetic energy of the left droplet (observed during the initial stage when it wets the surface) on the ellipsoid is much smaller than those on the concave and planar surfaces. This is not only because the attraction of the ellipsoidal wall to the droplet is the weakest (due to its particular shape), but also because the interaction between the two droplets on the ellipsoid has always been relatively large (as no separation occurs), and the left droplet is retarded by the right droplet the most. On the other hand, the maximum kinetic energy of the right droplet (observed after the initial stage) on the ellipsoid is the largest and occurs the earliest among all three cases. The reason is as follows. Because the compound droplet on the ellipsoid does not split, the right droplet is always in an accelerated state in the early stage of the wetting process. In contrast, for the planar and concave walls, the right droplet accelerates first, then decelerates after separation, and then accelerates again after the two droplets reconnect. For all three walls, the kinetic energies of both droplets become very low after a long time (e.g., t>400), as seen in Figure 6.

**Figure 7:**
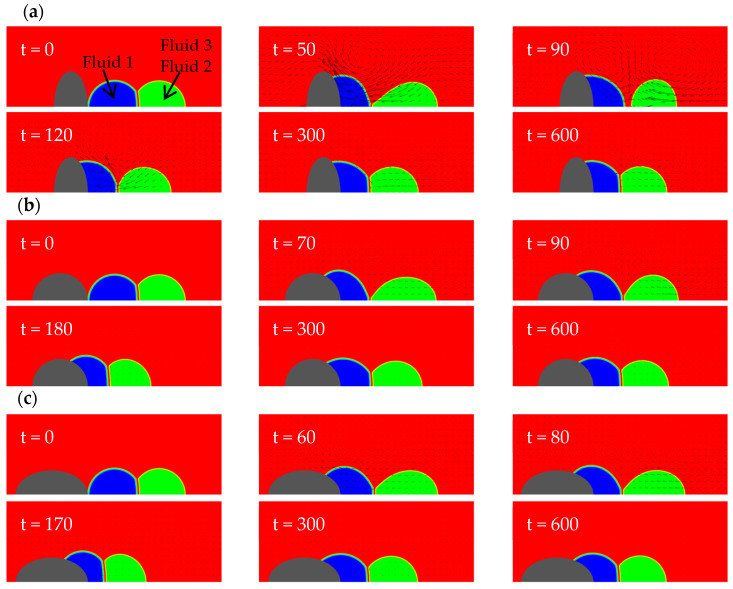
Evolution of the flow field of compound droplets spreading on ellipsoids with different curvatures, (**a**) $l_{x}=0.6$, $l_{y}=1.291$; (**b**) $l_{x}=l_{y}=1$; (**c**) $l_{x}=1.310$, $l_{y}=0.8736$.

As seen from the above, it is more difficult to make the compound droplet separate on an ellipsoidal wall than on a concave wall or a planar wall. Next, we further explore the wetting of a Janus droplet on ellipsoidal walls with different curvatures. Three different ellipsoids with the same volume (4.189) were studied. The first is an oblate spheroid with $l_{x}=0.6$, $l_{y}=1.291$. The second is a unit sphere. The third is the prolate spheroid just studied previously. The interfacial angles are slightly different from those in the above: $\varphi_{1}=155{}^{\circ}$, $\varphi_{2}=135{}^{\circ}$, $\varphi_{3}=70{}^{\circ}$. The other parameters are the same as those in Figure 3c. The flow field evolutions for the three cases are shown in Figure 7.

As seen in Figure 7, droplet separation only occurs near the oblate spheroid. During its spreading on the unit sphere and on the prolate spheroid, the left droplet is always connected with the right one. On the unit sphere, the interfacial area between the two droplets shrinks to a quite small value, from t=70 to t=90, approaching the critical state of droplet separation. However, at that critical moment, the distance between the two droplets reaches its maximum and the deformations of the interfaces also reach the largest degree. Thus, there is no further bending or distortion of the interface between the two droplets to make them disconnect. The situation on the prolate spheroid is similar to that in the above. These observations further confirm the importance of the second mechanism (the deformation induced separation). The oblate spheroid extends away from the axis the most among all three solid objects of the same volume and provides the largest area for the left droplet to spread upwards, leading to sufficiently large distortion of the left droplet in a short enough time. These are key to breaking up the Janus droplet.

### 3.4. Effects of the Radius of Curvature and Contact Angle of the Wall on Droplet Separation

**Figure 8:**
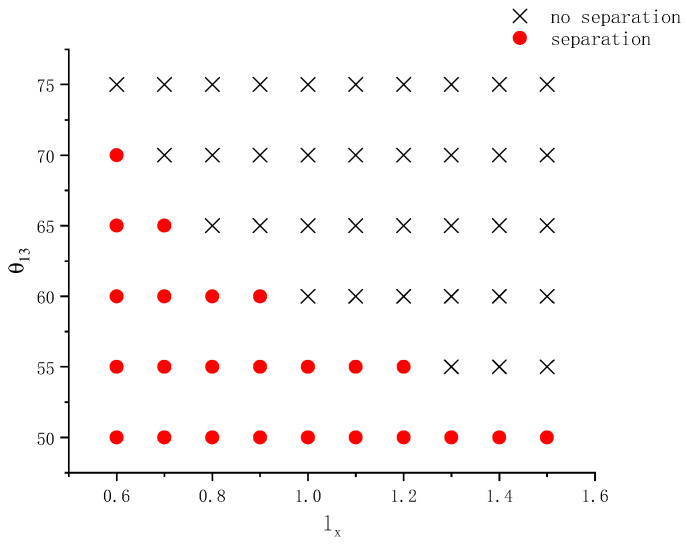
Phase diagram on whether a compound droplet separates during wetting and spreading on an ellipsoid having different shapes and contact angles.

This section explores the effects of the radius of curvature of the wall together with its wettability on the separation behavior of compound droplets over a wider parameter range. Both an ellipsoidal wall with variable positive curvature and a concave wall with a constant negative curvature were considered. The ellipsoid volume is fixed at 4.189. Several lengths of the semi-axis in the x-direction $l_{x}=0.7$, 0.8, 0.9, 1, 1.1, 1.2, 1.3 were tested. The corresponding values of $l_{y}$ are $l_{y}=1.195$, 1.118, 1.054, 1, 0.9535, 0.9129, and 0.8771, respectively. The contact angle $\theta_{13}$ varies from 50° to 75°. Other parameters are the same as those used in Figure 7 in Section 3.3. Figure 8 shows the phase diagram of the separation state on ellipsoids with different $l_{x}$ and $\theta_{13}$. In this figure, the cross symbol means no separation occurs and the filled circle means the contrary. With the decrease of $l_{x}$ (concurrently the increase of $l_{y}$), the radius of curvature of the ellipsoid near the axis ($r_{c}$) increases. The corresponding values of $r_{c}$ are $r_{c}=2.040$, 1.562, 1.234, 1, 0.8265, 0.6945, and 0.5918, respectively. From Figure 8 it can be seen that smaller contact angles are conducive to droplet separation. This is because more hydrophilic walls have a stronger attraction force on the left droplet, making its acceleration and deformation during the wetting process larger. In addition, at each contact angle, to decrease $l_{x}$ facilitates the occurrence of droplet separation. As the contact angle increases, the critical $l_{x}$ for droplet separation decreases. It means that to observe droplet separation during the spreading on a less hydrophilic ellipsoid, one must increase the radius of curvature. All these agree with previous findings and analyses.

**Figure 9:**
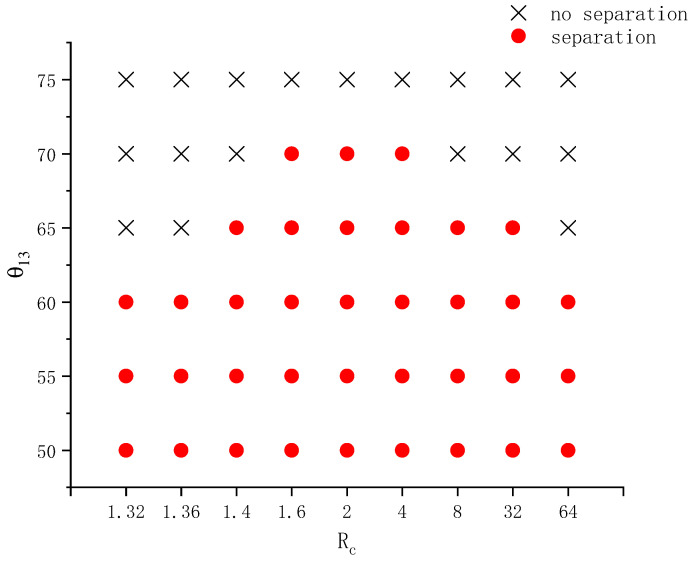
Phase diagram on whether a compound droplet separates during wetting and spreading on concave walls having different radii of curvature and contact angles.

In addition to ellipsoidal walls, we also varied the radius of curvature for a concave wall having a constant curvature. Based on previous results, droplet separation is more likely to occur on a concave wall. To encompass a broader range of parameters, the interfacial angles in this part are chosen as ${\;\varphi }_{1}=150{}^{\circ}$, $\varphi_{2}=130{}^{\circ}$, and $\varphi_{3}=80{}^{\circ}$. The contact angle varies from 50° to 75°. The radius of curvature changes from 1.32 to 64. Other parameters are not altered. The phase diagram of the separation state on a concave wall under different combinations of $R_{c}$ and $\theta_{13}$ are shown in Figure 9. Note that the increments in $R_{c}$ are nonuniform and the horizontal coordinates are not to scale. As found from this figure, when the wall is not so hydrophilic (e.g., $\theta_{13}=65{}^{\circ}$ and 70°) and the radius of curvature is large (e.g., $R_{c}>2$), one can see a transition from “no separation” to “separation” by properly reducing the radius of curvature. However, this effect of reducing $R_{c}$ fails to work when $R_{c}\leq 1.4$. At relatively small $R_{c}$, the volume enclosed by the concave wall is also small, and there is not enough space to accommodate the left droplet spreading outwards (i.e., away from the axis). When the left droplet does not have enough deformation, its connection with the right droplet is hard to break. Therefore, if one wants to change the radius of curvature of a concave wall to promote the separation of Janus droplets, a moderate value for $R_{c}$ should be taken.

### 3.5. Effects of the Interfacial Angles and Wall Contact Angle on Droplet Separation

**Figure 10:**
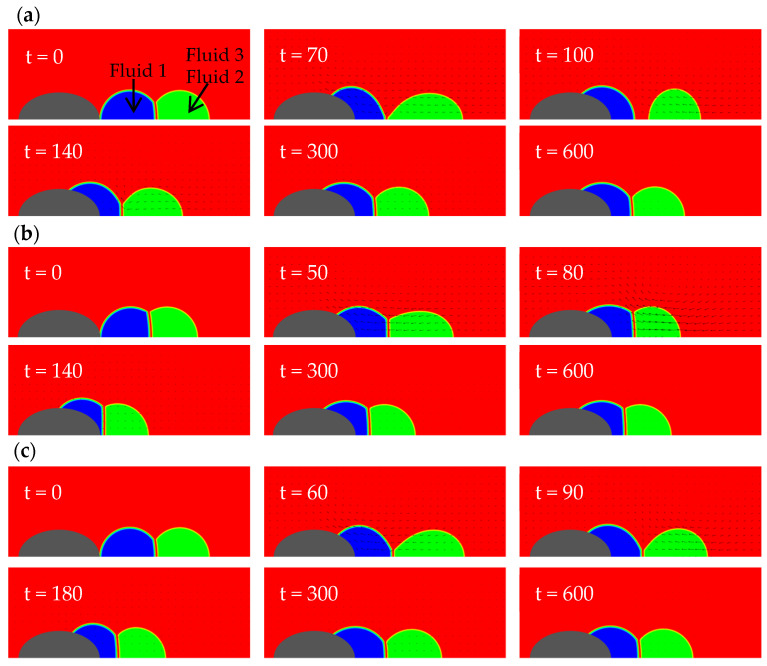
Evolution of the flow field of a compound droplet spreading on an ellipsoid under different interfacial angles: (**a**) ${\;\varphi }_{1}=155{}^{\circ}$, $\varphi_{2}=135{}^{\circ}$, $\varphi_{3}=70{}^{\circ}$, $\theta_{13}=50{}^{\circ}$; (**b**) ${\;\varphi }_{1}=135{}^{\circ}$, $\varphi_{2}=115{}^{\circ}$, $\varphi_{3}=110{}^{\circ}$, $\theta_{13}=50{}^{\circ}$; (**c**) ${\;\varphi }_{1}=155{}^{\circ}$, $\varphi_{2}=135{}^{\circ}$, $\varphi_{3}=70{}^{\circ}$, $\theta_{13}=60{}^{\circ}$.

In addition to the geometrical factors and wettability of the wall, the interfacial angles may also influence the droplet separation behavior. Figure 10a,b shows two typical wetting and spreading processes on a prolate spheroid with ${\;\varphi }_{1}=155{}^{\circ}$, $\varphi_{2}=135{}^{\circ}$, $\varphi_{3}=70{}^{\circ}$ and ${\;\varphi }_{1}=135{}^{\circ}$, $\varphi_{2}=115{}^{\circ}$, $\varphi_{3}=110{}^{\circ}$. The contact angle $\theta_{13}$ is fixed at $\theta_{13}=50{}^{\circ}$ for these two cases. In Figure 10c, the contact angle $\theta_{13}$ is $\theta_{13}=60{}^{\circ}$, and the interfacial angles are the same as in Figure 10a. All other parameters are the same as in Figure 3c. As found in Figure 10a, when ${\;\varphi }_{1}=155{}^{\circ}$, $\varphi_{2}=135{}^{\circ}$, $\varphi_{3}=70{}^{\circ}$, the two droplets separated for a period of time. In Figure 10b, when ${\;\varphi }_{1}=135{}^{\circ}$, $\varphi_{2}=115{}^{\circ}$, $\varphi_{3}=110{}^{\circ}$, the two droplets are always tightly connected throughout the wetting process. The significance of the interfacial angles may be demonstrated through the energy difference between two static states: one is the initial state and the other is an imaginary state (labelled with a superscript ${\;}^{sep}$) in which the left and right droplets are two separate free spheres. For the time being, we assume that no wall is present. Then, the system energy for the former state is $E_{t}^{0}=\sigma_{12}A_{12}^{0}+\sigma_{13}A_{13}^{0}+\sigma_{23}A_{23}^{0}$ and that for the latter is $E_{t}^{sep}=\sigma_{13}A_{13}^{sep}+\sigma_{23}A_{23}^{sep}$ with $A_{13}^{sep}=A_{23}^{sep}=4\pi R_{0}^{2}$. The scaled energy difference is found to be $\Delta E_{t}^{0\to sep}/\sigma_{12}=-0.1091$ for ${\;\varphi }_{1}=155{}^{\circ}$, $\varphi_{2}=135{}^{\circ}$, $\varphi_{3}=70{}^{\circ}$ and $\Delta E_{t}^{0\to sep}/\sigma_{12}=-0.8599$ for ${\;\varphi }_{1}=135{}^{\circ}$, $\varphi_{2}=115{}^{\circ}$, $\varphi_{3}=110{}^{\circ}$. From the energy perspective, the minimum energy required to split the Janus droplet for $\varphi_{1}=155{}^{\circ}$, $\varphi_{2}=135{}^{\circ}$, $\varphi_{3}=70{}^{\circ}$ is much smaller than that for $\varphi_{1}=135{}^{\circ}$, $\varphi_{2}=115{}^{\circ}$, $\varphi_{3}=110{}^{\circ}$. This explains the differences between Figure 10a,b. By comparing Figure 10a,c, it can be found that the wetting behavior at $\theta_{13}=50{}^{\circ}$ is obviously different from that under the same interfacial angles at $\theta_{13}=60{}^{\circ}$. This is due to the more hydrophilic wall’s stronger attraction, as stated before.

**Figure 11:**
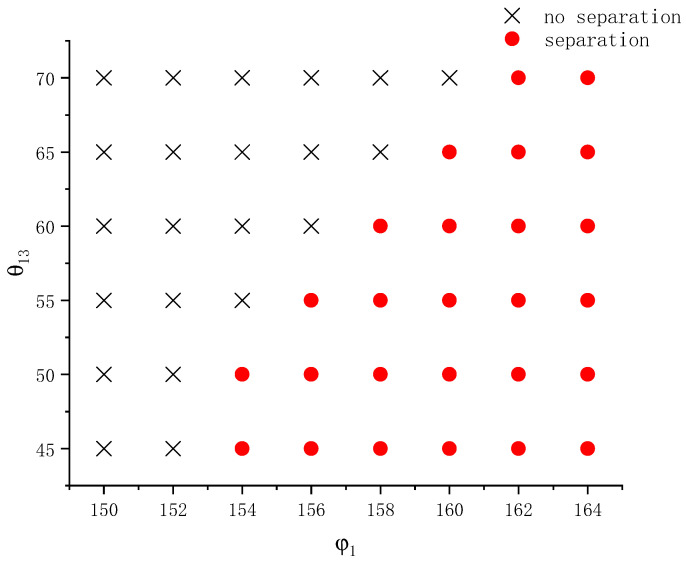
Phase diagram on whether a compound droplet separates during wetting and spreading on an ellipsoid at different interfacial angles and contact angles.

To know the effects of the interfacial angles and wall wettability on droplet separation in a broader parameter regime, a number of simulations on compound droplet wetting on the above prolate spheroid under different interfacial angles and contact angles were carried out. The interfacial angle $\varphi_{1}$ varies from 150° to 164°, and the contact angle $\theta_{13}$ varies from 45° to 70°. Note that the difference between the first two interfacial angles ($\varphi_{1}-\varphi_{2}$) is fixed at 20°. Other parameters are the same as those given before. Based on the simulation outcome, a phase diagram on whether droplet separation occurs during the wetting process is established in Figure 11. As shown in Figure 11, in general when the interfacial angle $\varphi_{1}$ increases, the critical contact angle for separation increases. For instance, at $\varphi_{1}=154{}^{\circ}$, the critical contact angle is between 50° and 55° and at $\varphi_{1}=158{}^{\circ}$ it is between 60° and 65°. These results indicate that, at a larger interfacial angle $\varphi_{1}$, the bond between the left and right droplets weakens and may be broken by the wetting on a less hydrophilic wall. Besides, the different variation ranges of the interfacial angle and contact angle in Figure 11 suggest that the interfacial angle has greater impact on the droplet separation phenomenon than the contact angle. While the separation phenomenon depends on both the intrinsic properties of both a Janus droplet, like the interfacial angles, and external factors, like the wall wettability, it seems that the former plays a more influential role than the latter.

**Figure 12:**
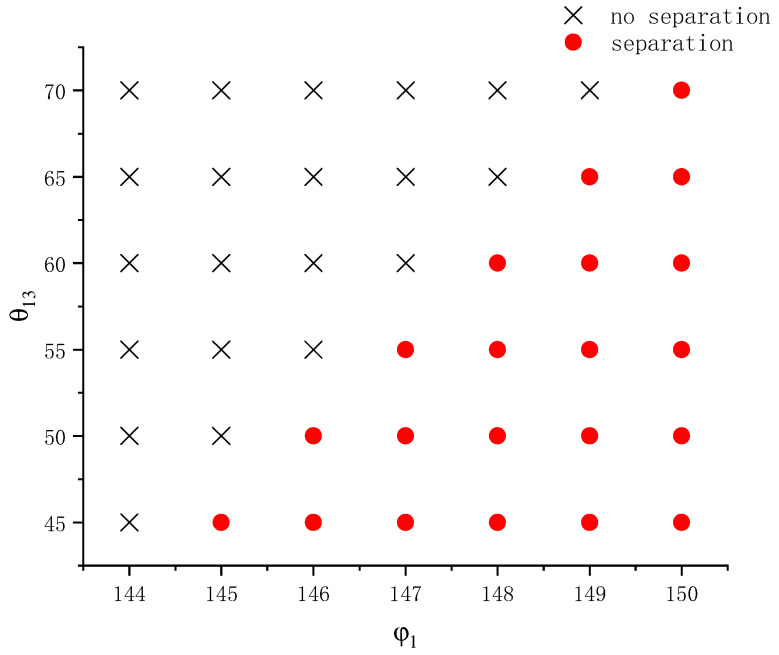
Phase diagram on whether a compound droplet separates during wetting and spreading on a concave surface at different interfacial angles and contact angles.

In addition to a prolate spheroid, we also performed similar investigations for a concave wall with a radius of curvature $R_{c}=4$ and generated a phase diagram in the $\varphi_{1}-\theta_{13}$ plane under the same physical conditions, as shown in Figure 12. Like Figure 11, the range of variation for the contact angle is also from 45° to 70°. As found from Figure 11 and Figure 12, when other parameters are equal, the interfacial angle $\varphi_{1}$ required for the separation of compound droplets on the concave wall is smaller than that on the prolate spheroid. As mentioned above, a smaller interfacial angle $\varphi_{1}$ corresponds to a stronger bond between the two droplets. This indicates that under the same wall wettability, the concave surface drags and deforms the left droplet more severely than the convex surface (such as the ellipsoid). This agrees with previous analyses in Section 3.3. By comparing the variation ranges of the interfacial angle in Figure 11 and Figure 12, it can be seen that the influence of the interfacial angle on droplet separation is even more significant on the concave surface than on the ellipsoid. For example, on the concave surface at $\varphi_{1}=146{}^{\circ}$, the critical contact angle for droplet separation is between 50° and 55° and at $\varphi_{1}=148{}^{\circ}$ it is between 60° and 65°. Roughly speaking, to have a ~10° change in the critical contact angle, it takes only a ~2° change in $\varphi_{1}$ on the concave surface, whereas it requires a ~4° change in $\varphi_{1}$ on the ellipsoid. These findings indicate that the influence of the interfacial angle $\varphi_{1}$ is amplified to some extent by the concave surface. In other words, the geometry of the wall not only affects whether droplet separation happens under the same wettability, but also affects the sensitivity of this phenomenon to the interfacial angle $\varphi_{1}$. The stronger sensitivity to the interfacial angle $\varphi_{1}$ on the concave wall can be understood from the following perspective. Because in the early state the left droplet experiences more extensive spreading and larger deformation on the concave wall, the interfacial area between the two droplets shrinks quickly to a low value (e.g., see Figure 3a in Section 3.3). Under such circumstances, even a seemingly minor change in the interfacial angle $\varphi_{1}$ can determine whether it will further shrink to zero or not.

The above findings from Figure 12 may also be understood from the other side. That is, given the same change in the interfacial angle, the influence of the contact angle is smaller on the concave surface than on the ellipsoidal wall. This is because the wetting effect of the concave solid wall itself on the droplets has been relatively good, and the influence of continuing to enhance its wetting effect on the separation of droplets will not be too great. On the other hand, it can be seen from Figure 3a in Section 3.3 that when the droplets on the concave surface are separated, droplet 1 has been wetted on the wall to a considerable extent. The possibility of separation mainly depends on whether the liquid bridge between two droplets will break when droplet 1 spreads to the maximum on the solid wall; this condition is closely related to the interfacial angle, so the interfacial angle is the most influential factor in the process of droplet separation on the concave surface whose $R_{c}=4$.

### 3.6. Effect of the Density Ratio of Droplet to Ambient Fluid on Droplet Separation

**Figure 13:**
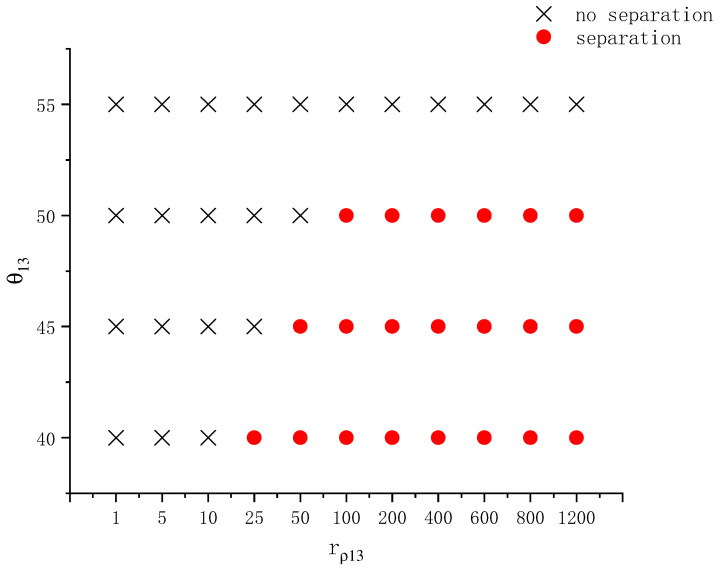
Phase diagram on whether a compound droplet separates during wetting and spreading on a prolate spheroid at different density ratios and contact angles.

In addition to the wall properties and interfacial angles, the density ratio may also have certain impacts on the wetting behavior of a compound droplet. This section explores the influence of the density ratio of droplet to ambient fluid ($r_{\rho 13}$) on the separation behavior of Janus droplets. The interfacial angles are fixed at $\varphi_{1}=155{}^{\circ}$, $\varphi_{2}=135{}^{\circ}$, $\varphi_{3}=70{}^{\circ}$. The same prolate spheroid as above is used. In addition to $r_{\rho 13}$, the contact angle $\theta_{13}$ is also varied. Other parameters are the same as those given in Section 3.3. Note that the kinematic viscosity ratio $r_{\upsilon 13}$ is fixed at 0.06. Thus, as the density ratio $r_{\rho 13}$ changes, the dynamic viscosity of the ambient fluid varies. Based on the simulation results using different combinations of $r_{\rho 13}$ and $\theta_{13}$, another phase diagram on droplet separation on an ellipsoidal wall is established in Figure 13. From this figure, one can see that, in general, a larger density ratio $r_{\rho 13}$ is favorable for droplet separation at a given contact angle $\theta_{13}$. A larger density ratio $r_{\rho 13}$ corresponds to a lighter ambient fluid. The dynamic viscosity of the ambient fluid is also smaller at a higher $r_{\rho 13}$ (with $r_{\upsilon 13}$ being the same). These two factors reduce the obstruction of the ambient fluid to the motion of the compound droplet, making droplet separation easier. From the results at $\theta_{13}=55{}^{\circ}$, it is observed that when the contact angle is not small enough, droplet separation will not occur even if the density ratio is very large.

## 4. Conclusions

To conclude, the wetting and spreading behavior of a Janus droplet on solid walls of various shapes and wettabilities was investigated by axisymmetric LBM simulations, mostly at high density ratios. The evolutions of the interfaces and interfacial energies were examined to determine whether the two constituent droplets separate during the wetting process. The main factors found to promote the occurrence of droplet separation include (a) a concave wall with moderate radius of curvature, (b) a convex wall with large radius of curvature, (c) a small contact angle for the droplet near the wall, (d) large interfacial angles in the droplets, and (e) a large density ratio of the droplet to ambient fluid.

Comparisons between the wetting processes on three types of walls suggest that the moderately outward extending concave wall has better apparent wettability and enhances the spreading speed of the wetting droplet, leading to its large deformation and large velocity difference between the two constituent droplets. In the study of solid walls of various spheroid shapes, it was found that the larger the radius of curvature near the wetting droplet is, the faster its contact line moves on the wall, and the two droplets are more likely to separate. The influence of curvature for concave walls is non-monotonic. Only when the curvature is small, droplet separation is more likely to occur with its increase. When the curvature increases to a certain extent, the small radius of curvature limits the spreading of the wetting droplet and prevents droplet separation. When the contact angle of the wetting droplet is smaller, it experiences stronger attraction forces towards the wall and the distance between the two droplets increases more rapidly. As the interfacial angles in the constituent droplets increase, less energy is needed to split the compound droplet. When the density of the ambient fluid decreases, the compound droplet is less hindered, and the wetting droplet is allowed to move faster during its spreading. The five phase diagrams on droplet separation state, based on numerous simulations at different conditions, clearly show the influence of various factors for different walls. These results and findings may be helpful to future studies of Janus droplets near solid walls and could also advance the understanding of the interaction between compound droplets and solid microparticles. In addition, the study of the equilibrium morphology of compound droplets on curved surfaces might find applications in microfabrication of certain small components with special shapes (e.g., microlens).

Finally, the present work has some limitations. First, this study only discussed the wetting and spreading of compound droplets on curved surfaces under axisymmetric settings. To capture the behavior of compound droplets in many real situations, full three-dimensional simulations may be necessary. Second, the droplet separation reported in this work is only transient. It requires further investigations on how to sustain the separation of two constituent fluids.

## Figures and Tables

**Figure A1:**
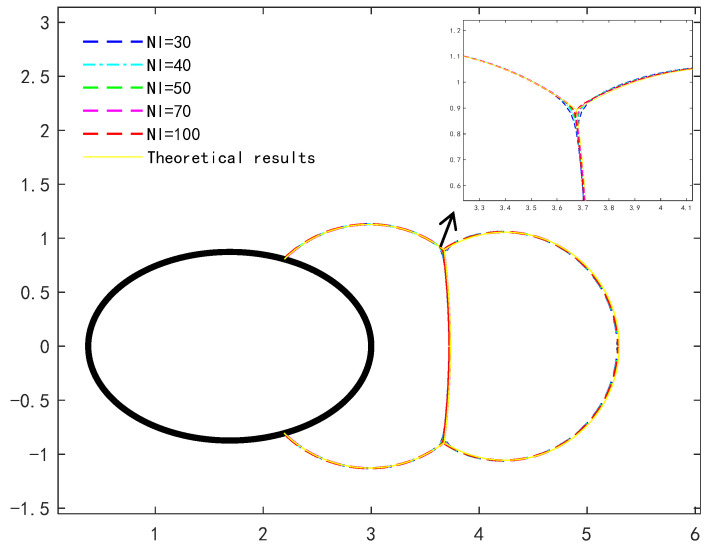
Equilibrium shapes of a compound droplet on a prolate spheroid obtained under different grid densities.

**Figure A2:**
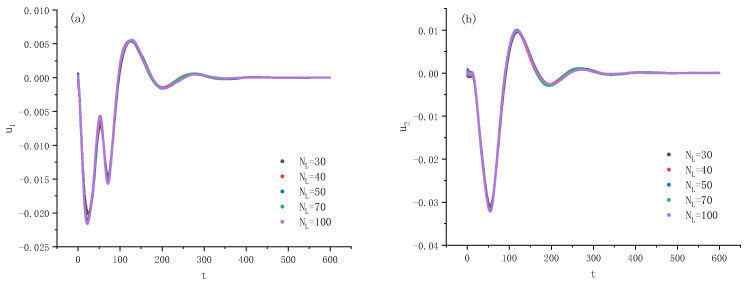
The evolutions of the velocity of the centroids of the two droplets along the x-axis under different grid densities: (**a**) the centroid velocity of the left droplet $u_{1}$; (**b**) the centroid velocity of the right droplet $u_{2}$.

**Figure A3:**
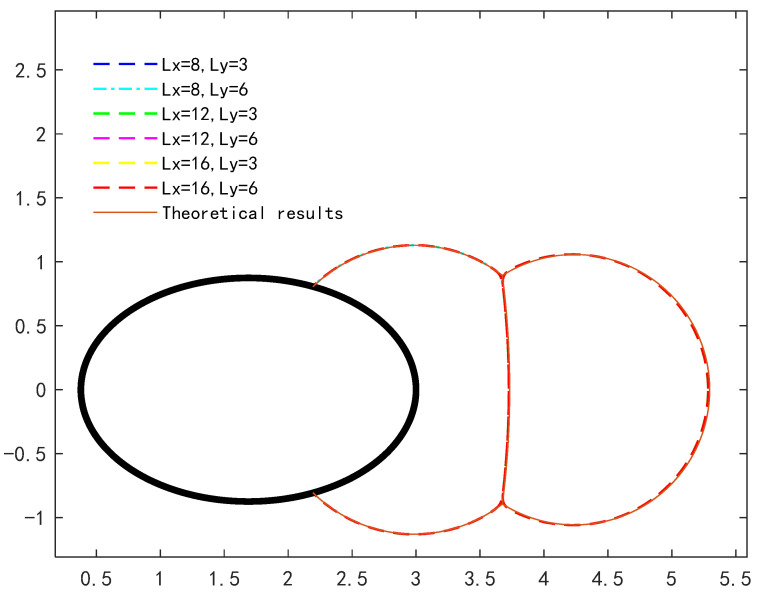
Equilibrium shapes of the compound droplet obtained by using different computational domains.

**Figure A4:**
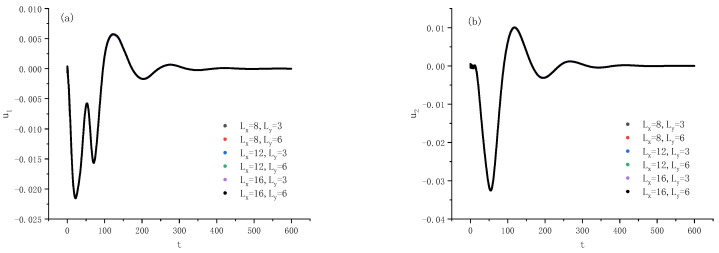
The evolutions of the velocity of the centroids of the two droplets along the x-axis obtained by using different computational domains: (**a**) the centroid velocity of the left droplet $u_{1}$; (**b**) the centroid velocity of the right droplet $u_{2}$.

**Figure A5:**
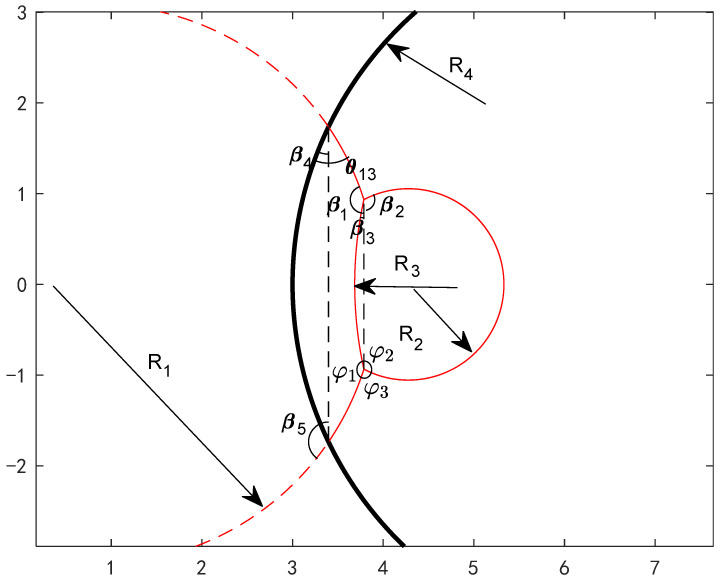
Equilibrium configuration of a compound droplet on a concave surface with negative curvature.

**Figure A6:**
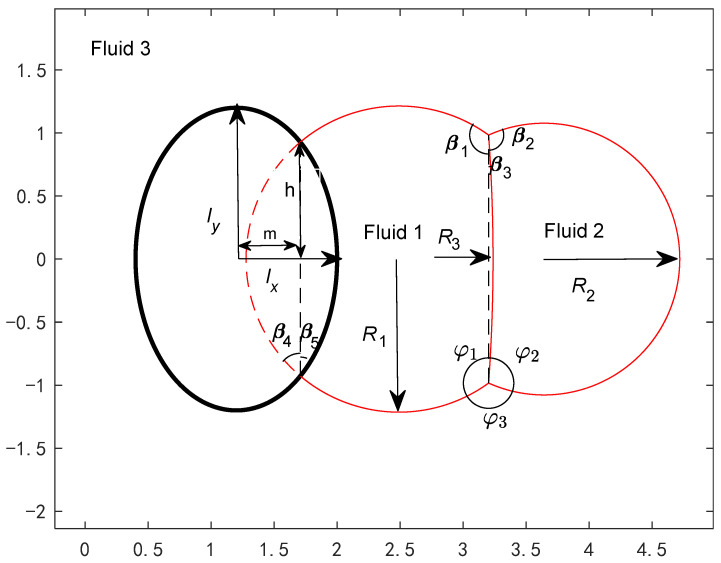
Equilibrium configuration of a compound droplet on an oblate spheroid.

## Data Availability

The data that support the findings of this study are available from the corresponding author upon reasonable request.

## Appendix A. Some Details of the Numerical Method

### Appendix A.1. Some Details for the Axisymmetric LBEs for the CACEs

In Equation (5), the matrix M is given by

(A1) $$M=\left[\begin{array}{ccccccccc} 1 & 1 & 1 & 1 & 1 & 1 & 1 & 1 & 1\\ -4 & -1 & -1 & -1 & -1 & 2 & 2 & 2 & 2\\ 4 & -2 & -2 & -2 & -2 & 1 & 1 & 1 & 1\\ 0 & 1 & 0 & -1 & 0 & 1 & -1 & -1 & 1\\ 0 & -2 & 0 & - & 0 & 1 & -1 & -1 & 1\\ 0 & 0 & 1 & 0 & -1 & 1 & 1 & -1 & -1\\ 0 & 0 & -2 & 0 & 2 & 1 & 1 & -1 & -1\\ 0 & 1 & -1 & 1 & -1 & 0 & 0 & 0 & 0\\ 0 & 0 & 0 & 0 & 0 & 1 & -1 & 1 & -1 \end{array}\right]$$

$M^{-1}$ is its inverse that transforms the moments back into the DFs, $S_{g}=diag(1,1,1,s_{g},s_{g},s_{g},s_{g},1,1)$ is the diagonal relaxation matrix with $s_{g}=1/\tau_{g}$ and $\tau_{g}=0.5+m_{0}/\left(c_{s}^{2}\delta_{t}\right)$ and $G_{l}(\vec{x},t)$ is a source term which can be expressed as,

(A2) $$G_{l}={{\left[M^{-1}({I-0.5S}_{g})M\right]}_{lm}R}_{m},$$

with I being the unit matrix and $R_{m}=\frac{1}{c_{s}^{2}}w_{m}e_{m\alpha }[\partial_{t}(rc_{1}u_{\alpha }+m_{0}c_{1}\delta_{\alpha r})+c_{s}^{2}r(\lambda_{1}n_{1,\alpha }-\beta_{1,\alpha })]$. Here $c_{s}$ is the “speed of sound” in LBM and for the D2Q9 velocity model $c_{s}=\frac{c}{\sqrt{3}}$ ($c=\frac{\delta_{x}}{\delta_{t}}$ is the lattice velocity and $\delta_{x}$ is the grid size). The moments of the source term $R_{m}$ is found to be ${\left[0,0,0,q_{1},-q_{1},q_{2},-q_{2},0,0\right]}^{T}$ where the terms $q_{1}$ and $q_{2}$ are given by $q_{1}=\partial_{t}(rc_{1}u_{z}){+c}_{s}^{2}r(\lambda_{1}n_{1,z}-\beta_{1,z})$ and $q_{2}=\partial_{t}(rc_{1}u_{r}+m_{0}c_{1}){+c}_{s}^{2}r(\lambda_{1}n_{1,r}-\beta_{1,r})$. The equilibrium moments for $g_{m}^{eq}$ are given by ${\left[rc_{1},-2rc_{1},rc_{1},rc_{1}u_{z},-rc_{1}u_{z},rc_{1}u_{r}+m_{0}c_{1},-(rc_{1}u_{r}+m_{0}c_{1}),0,0\right]}^{T}$.

The boundary conditions for the volume fractions near solid walls inside the domain are implemented in the same way as in [22]. The 1st- and 2nd-order spatial derivatives of the volume fractions are evaluated by the 4th-order isotropic schemes at bulk nodes (i.e., computational nodes away from the solid boundary) [27],

(A3) $$\partial_{\alpha }c_{i}=\frac{1}{6c_{s}^{2}\delta_{t}}\sum_{l=1}^{8}w_{l}e_{l\alpha }[8c_{i}(\vec{x}+{\vec{e}}_{l}\delta_{t})-c_{i}(\vec{x}+2{\vec{e}}_{l}\delta_{t})],$$

(A4) $$\frac{\partial^{2}c_{i}}{\partial x^{2}}+\frac{\partial^{2}c_{i}}{\partial y^{2}}=\frac{1}{6c_{s}^{2}\delta_{t}^{2}}\sum_{l=1}^{8}w_{l}[16c_{i}(\vec{x}+{\vec{e}}_{l}\delta_{t})-c_{i}(\vec{x}+2{\vec{e}}_{l}\delta_{t})-15c_{i}(\vec{x})],$$

and by the 2nd-order isotropic schemes based on the D2Q9 lattice velocity model at boundary nodes (i.e., computational nodes near the solid boundary). Here $w_{l}$ is the weight for the direction along ${\vec{e}}_{l}$. Besides, the halfway bounce-back boundary condition is applied for $g_{l}$ and $h_{l}$ near all solid walls to ensure the conservation of volume fractions [28].

### Appendix A.2. Some Details for the Axisymmetric LBEs for the NSEs

To improve stability, the MRT collision model [21] was also used for the axisymmetric LBEs for the NSEs. The MRT model in the LBM for the NSEs is overall similar to that for the CACEs. There are some differences in the diagonal relaxation matrix which is changed to $S_{f}=diag(s_{0},\;s_{1},\;s_{2},\;s_{3},\;s_{4},\;s_{5},\;s_{6},\;s_{7},\;s_{8})$ with $s_{7}=s_{8}=s_{f}=1/\tau_{f}$ (to control the shear viscosity) and $s_{1}$ being a tunable parameter related to the bulk viscosity ($s_{1}$ = 1 was used to enhance the damping of undesirable acoustic waves and help alleviate the chequerboard effects) [21,29]. The remaining relaxation parameters only affect the simulation results marginally and may be set to unity. On an immersed solid boundary of any shape, the interpolated bounce-back method is applied for $f_{l}$ according to [30]. To suppress the chequerboard effect, in the streaming step the Lax-Wendroff streaming [31] is used for $f_{l}$,

(A5) $$f_{l}(\vec{x},t+\delta_{t})=\alpha_{0}f_{l}^{\prime }(\vec{x},t)+\alpha_{1}f_{l}^{\prime }(\vec{x}+{\vec{e}}_{l}\delta_{t},t)+\alpha_{-1}f_{l}^{\prime }(\vec{x}-{\vec{e}}_{l}\delta_{t},t),$$

where $f_{l}^{\prime }$ denotes the post-collision DF, and the coefficients are given by ${\;a}_{0}=1-A^{2}$, $\alpha_{-1}=A(A+1)/2$, $\alpha_{1}=A(A-1)/2$. (A=0.999 was used in our simulation). It is noted that to apply the Lax-Wendroff streaming only for $f_{l}$ is sufficient to suppress the chequerboard effect for most cases and the streaming of the DFs $g_{l}$ and $h_{l}$ (for the CACEs) is not changed (e.g., $g_{l}(\vec{x},t+\delta_{t})=g_{l}^{\prime }(\vec{x}-{\vec{e}}_{l}\delta_{t},t)$. It is also noted that the Lax-Wendroff streaming is used for $f_{l}$ at the bulk nodes only and the basic streaming is still used at the boundary nodes.

### Appendix A.3. The Chemical Potential and Interfacial Tension Force

In the above, it is seen that the CACEs do not involve the chemical potentials, but the interfacial tension force requires them. The chemical potential used in the calculation of the interfacial tension force $u_{c_{i},ST}$ is given by [22,32],

(A6) $$\mu_{c_{i},ST}=\frac{48\gamma_{i}}{W}c_{i}(c_{i}-1)(c_{i}-0.5)-\frac{3}{2}\gamma_{i}W\nabla^{2}c_{i},$$

where the phase specific interfacial tensions $\gamma_{i}(i=1,2,3)$ are given by $\gamma_{1}=\frac{1}{2}(\sigma_{12}+\sigma_{13}-\sigma_{23})$, $\gamma_{2}=\frac{1}{2}(\sigma_{12}+\sigma_{23}-\sigma_{13})$ and $\gamma_{3}$ $=\frac{1}{2}\left(\sigma_{13}+\sigma_{23}-\sigma_{12}\right)$ [10]. For convenience, we actually calculated a scaled chemical potential,

(A7) $${\tilde{\mu }}_{c_{i},ST}=\frac{\mu_{c_{i},ST}}{\gamma_{i}}=\frac{48}{W}c_{i}(c_{i}-1)(c_{i}-0.5)-\frac{3}{2}W\nabla^{2}c_{i},$$

Then, the interfacial tension force for ternary fluids can be calculated as,

(A8) $${\vec{F}}_{ST}=\sum_{i=1}^{3}\mu_{c_{i},ST}\nabla c_{i}=\sum_{i=1}^{3}{\gamma_{i}\tilde{\mu }}_{c_{i},ST}\nabla c_{i},$$

It is noted that in the axisymmetric formulation the Laplacian of $c_{i}$ in Equation (A6) must be modified as,

(A9) $$\nabla_{axisym}^{2}c_{i}=\frac{\partial^{2}c_{i}}{\partial z^{2}}+\frac{\partial^{2}c_{i}}{\partial r^{2}}+\frac{1}{r}\frac{\partial c_{i}}{\partial r}.$$

## Appendix B. Tests on Grid Density

The influence of grid density on the simulation results was investigated for one case of compound droplet spreading on a prolate spheroid. The common parameters used in the simulations are: $\theta_{13}=60{}^{\circ}$, $r_{\rho 12}=1$, $r_{\rho 13}=825$, $r_{\upsilon 12}=1$, $r_{\upsilon 13}=0.06$, Re=400. The interfacial angles are:${\;\varphi }_{1}=135{}^{\circ}$, $\varphi_{2}=115{}^{\circ}$, $\varphi_{3}=110{}^{\circ}$. The prolate spheroid has semi-axis lengths in the x-direction and y-direction $l_{x}=1.310$, $l_{y}=0.8736$. The domain size is selected $L_{x}\times L_{y}=8\times 3$. The following spatial discretization parameters were tested: $N_{L}=30$, 40, 50, 70, 100. The corresponding temporal discretization parameters were $N_{t}=120$, 214, 332, 655, 1332, respectively. The final equilibrium shapes of the compound droplet under different grid densities are shown in Figure A1. It can be seen from the figure that overall the results in equilibrium state obtained by using different grid densities are close and some small differences exit mainly near the three-phase point. Besides, the evolutions of the velocity of the two droplets’ centroids along the x- direction are shown in Figure A2. It can be found that the dynamic results obtained under different grids are overall similar as well. The evolutions of the droplets’ average velocity at $N_{L}=30$, 40 look slightly different from those at $N_{L}=50$, 70, 100 (mainly near the valleys in Figure A2). More careful examinations reveal that the maximum difference in the maximum magnitude of the centroid velocity of the left droplet between $N_{L}=50$ and $N_{L}=100$ is about 0.36%. Considering that the high grid density consumes a lot of computing resources and such difference occurs only over a short period of time, in the end $N_{L}=50$ was selected for the case studies in the main text.

## Appendix C. Tests on the Domain Size

For the dynamic problem of compound droplets spreading on a surface, the size of the simulation domain may affect the results. The influence of the computational domain on the results was investigated for the same case in Appendix B with $N_{L}=50$. Six different domain sizes were tested: $L_{x}\times L_{y}=8\times 3$, $L_{x}\times L_{y}=8\times 6$, $L_{x}\times L_{y}=12\times 3$, $L_{x}\times L_{y}=12\times 6$, $L_{x}\times L_{y}=16\times 3$, $L_{x}\times L_{y}=16\times 6$. The corresponding grid numbers are: $N_{x}\times N_{y}=400\times 150$, $N_{x}\times N_{y}=400\times 300$, $N_{x}\times N_{y}=600\times 150$, $N_{x}\times N_{y}=600\times 300$, $N_{x}\times N_{y}=800\times 150$, $N_{x}\times N_{y}=800\times 300$. Figure A3 shows the equilibrium shapes of the compound droplet obtained by using the above six domains. As observed from this figure, the results by using different domains almost overlap. Figure A4 shows the evolutions of the centroidal velocity along the x-axis of the two droplets during the wetting and spreading process. It can be seen that the dynamic results by simulations with different domains are also nearly the same. Thus, $L_{x}\times L_{y}=8\times 3$ was used in all simulations in the main text.

## Appendix D. Analytical Solution for the Shape of Compound Droplets on a Negative Curved Concave Surface

Figure A5 shows the equilibrium configuration of an axisymmetric compound droplet on a concave surface with a constant negative curvature with the relevant quantities illustrated. In the figure, the black solid line is the curved solid wall, the red solid lines are the droplet interfaces, the red dashed line is the extension of the interface between fluid 1 and fluid 3, and the black dashed lines perpendicular to the x-axis are drawn through two different pairs of three-phase points as auxiliary lines. The following parameters are already known:

1. The radius of curvature of the concave wall: $R_{4}$;
2. The initial volume of the two droplets: $\frac{4}{3}\pi R_{0}^{3}$;
3. The interfacial angles: $\varphi_{1},\;\varphi_{2},\;\varphi_{3}$;
4. The contact angle of the left droplet on the wall: $\theta_{13}$;

There are eight unknowns in total. The first three are the radius of curvature of the interface between the left droplet and the ambient fluid ($R_{1}$), the radius of curvature of the interface between the right droplet and the ambient fluid ($R_{2}$), and the radius of curvature of the interface between the two droplets ($R_{3}$). In addition, there are five angles formed by the two auxiliary lines and several tangent lines passing through the two different three-phase points (as illustrated in Figure A5):${\;\beta }_{1},\;\beta_{2},\beta_{3},\beta_{4},\beta_{5}$. Eight independent equations are employed to solve for them. The first two equations are obtained from the interfacial angles,

(A10) $$\varphi_{1}=\beta_{1}-\beta_{3},$$

(A11) $$\varphi_{2}=\beta_{2}+\beta_{3},$$

Then according to the geometric relationship between each two adjacent interfaces, one has,

(A12) $$R_{1}sin\beta_{1}=R_{2}sin\beta_{2},$$

(A13) $$R_{1}sin\beta_{1}=R_{3}sin\beta_{3},$$

(A14) $$R_{1}sin\beta_{5}=R_{4}sin\beta_{4},$$

From the contact angle condition, one has,

(A15) $$\theta_{13}=\pi +\beta_{4}-\beta_{5},$$

Finally, from the volume conservation conditions for the two droplets, one can obtain:

(A16) $$\pi R_{1}^{3}(2-3cos\beta_{1}+{cos\beta_{1}}^{3})/3-{\pi R}_{3}^{3}(2-3cos\beta_{3}+{cos\beta_{3}}^{3})/3+{\pi R}_{4}^{3}(2-3cos\beta_{4}+{cos\beta_{4}}^{3})/3-{\pi R}_{1}^{3}(2-3cos\beta_{5}+{cos\beta_{5}}^{3})/3=4\pi R_{0}^{3}/3,$$

(A17) $${\pi R}_{2}^{3}(2-3cos\beta_{2}+{cos\beta_{2}}^{3})/3+\pi R_{3}^{3}(2-3cos\beta_{3}+{cos\beta_{3}}^{3})/3=4\pi R_{0}^{3}/3,$$

The analytical solution of this problem can be obtained by solving the above eight equations using Newton’s method (for brevity the details are omitted).

## Appendix E. Analytical Solution for the Shape of Compound Droplets on an Oblate Spheroid

Figure A6 shows the equilibrium configuration of an axisymmetric compound droplet on an oblate spheroid with the relevant quantities illustrated. The lines are shown in a way similar to that in Appendix D. The known parameters are almost the same as those in Appendix D except that for the oblate spheroid the semi-axis lengths ($l_{x}$ and $l_{y}$) are given. For convenience, introduce an auxiliary variable $m=\sqrt{l_{x}^{2}-l_{x}^{2}h^{2}/l_{y}^{2}}$, where h is the distance from the contact line of the left droplet on the wall to the axis. With h and m included, there are ten unknowns in total (the other eight are the same as those in Appendix D). The solutions of them require ten independent equations. First, since the contact line is on the oblate spheroid, one can find two relations for h, m and the angle $\beta_{5}$ from the equation for the oblate spheroid as,

(A18) $$m=\sqrt{l_{x}^{2}-l_{x}^{2}h^{2}/l_{y}^{2}},$$

(A19) $${\left(\frac{R_{4}x}{R_{4}y}\right)}^{2}\frac{h}{m}=tan\beta_{5},$$

Then the remaining eight equations are similar to those in Appendix D:

(A20) $$\varphi_{1}=\beta_{1}+\beta_{3},$$

(A21) $$\varphi_{2}=\beta_{2}-\beta_{3},$$

(A22) $$R_{1}sin\beta_{1}=R_{2}sin\beta_{2},$$

(A23) $$R_{1}sin\beta_{1}=R_{3}sin\beta_{3},$$

(A24) $$h=R_{1}sin\beta_{4},$$

(A25) $$\theta_{13}=\pi -\beta_{4}-\beta_{5}$$

(A26) $$\pi R_{1}^{3}(2-3cos\beta_{1}+{cos\beta_{1}}^{3})/3+{\pi R}_{3}^{3}(2-3cos\beta_{3}+{cos\beta_{3}}^{3})/3-{\pi R}_{1}^{3}(2-3cos\beta_{4}+{cos\beta_{4}}^{3})/3-\frac{2\pi }{3}{R_{4}x}^{2}(R_{4}x-m)=4\pi R_{0}^{3}/3,$$

(A27) $${\pi R}_{2}^{3}(2-3cos\beta_{2}+{cos\beta_{2}}^{3})/3-\pi R_{3}^{3}(2-3cos\beta_{3}+{cos\beta_{3}}^{3})/3=4\pi R_{0}^{3}/3,$$

Note that Equations (A20), (A21), (A26) and (A27) are slightly different from those in Appendix D because the definition of $\beta_{3}$ is different (the direction for positive $\beta_{3}$ is the opposite to the above). In Equation (A26) the last term on the left side represents the volume of the part of the oblate spheroid on the right side of the contact line. Finally, the analytical solution of this problem is obtained by solving the above ten equations using Newton’s method.
